# Machine learning approach for enumeration of circulating cells with diffuse *in vivo* flow cytometry

**DOI:** 10.1117/1.BIOS.3.3.032903

**Published:** 2026-09-04

**Authors:** Mehrnoosh Emamifar, Jane Lee, Malcolm Shumel, Joshua Pace, Chiara Bellini, Mark Niedre

**Affiliations:** Northeastern University, Department of Bioengineering, Boston, Massachusetts, United States

**Keywords:** circulating tumor cells, diffuse *in vivo* flow cytometry, machine learning, convolutional neural network, signal processing, cancer detection

## Abstract

**Significance:**

Diffuse *in vivo* flow cytometry (DiFC) is an emerging technique for enumerating rare, fluorescently labeled circulating tumor cells (CTCs) in small animals without drawing blood samples. DiFC uses detection of transient fluorescent peaks in time-series data. Previously, we used a simple amplitude threshold-based method for identifying peak candidates, but this ignored potentially useful information in peak shape that could reduce false-positive detections and increase detection efficiency of lower-amplitude peaks.

**Aim:**

Our aim is to develop a machine learning (ML)-integrated signal processing approach for DiFC that improves CTC enumeration by better discriminating CTC peaks from artifacts.

**Approach:**

We developed an ML-integrated approach that incorporates a convolutional neural network (CNN) classifier. The CNN was trained to distinguish CTC peaks from artifacts by analyzing peak amplitude and temporal shape characteristics. Performance was evaluated on *in silico*, control, and CTC-bearing mouse datasets.

**Results:**

The CNN classifier achieved accuracy, precision, sensitivity, and specificity exceeding 94% on test data. Compared with our previously published threshold-based approach, the ML-integrated method increased the number of correctly identified CTCs and their flow direction while reducing false detections across evaluation datasets.

**Conclusions:**

The ML-integrated approach substantially improves DiFC CTC enumeration, enabling robustness against artifacts in noisy conditions.

Statement of Discovery This work utilizes diffuse *in vivo* flow cytometry (DiFC) integrated with a convolutional neural network classifier to distinguish true circulating tumor cell signals from artifacts in fluorescence time-series data. The machine-learning-based approach achieves greater than 94% accuracy, sensitivity, and specificity on test data, improving DiFC circulating tumor cell enumeration with increased robustness against artifacts in noisy conditions.

## 1 Introduction

**Fig. 1:**
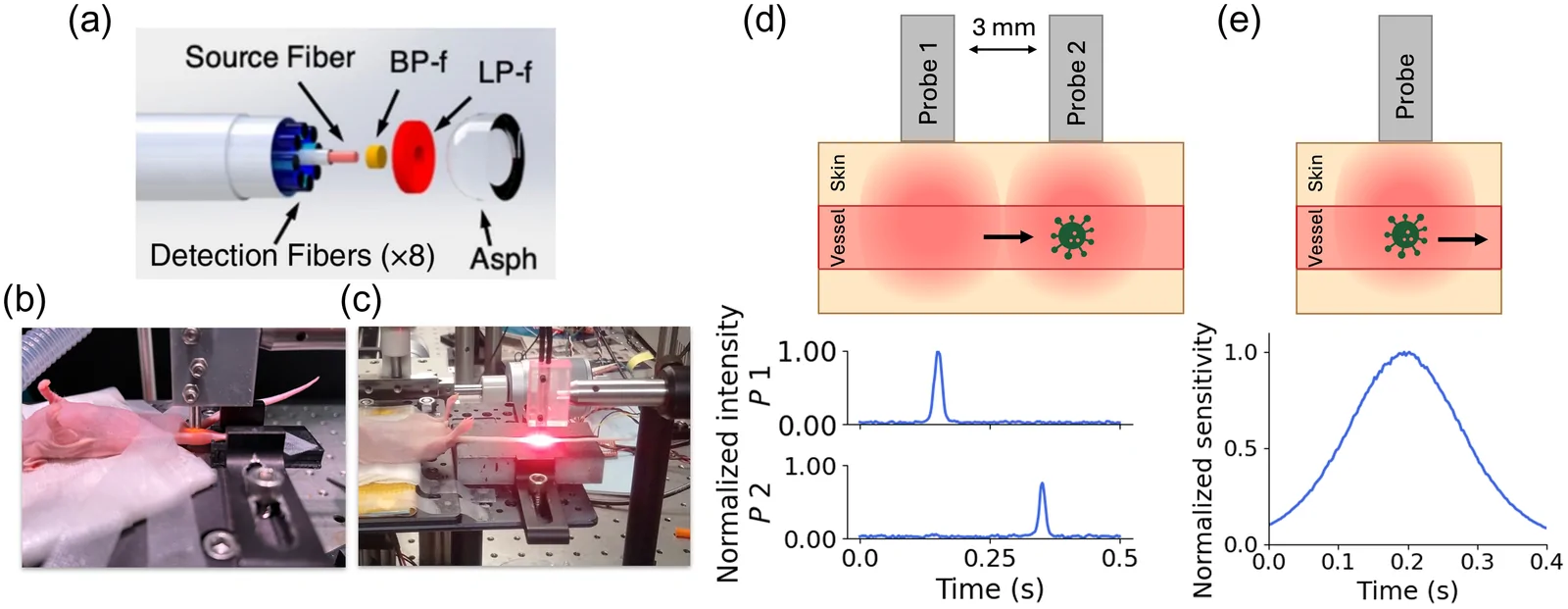
DiFC instrumentation. (a) Diagram of a DiFC probe, reproduced with permission from Ref. 6. We normally perform DiFC on the (b) hindlimb or (c) tail of mice. (d) Two DiFC probes are placed along the blood vessel, which permits detection of fluorescently labeled cells with a time delay corresponding to the cell speed. (e) Peaks have a characteristic shape which is defined by the sensitivity function of the DiFC probe. An example peak shape (computed with a Monte Carlo simulation^14^) for 0.75 mm depth cells is shown.

Metastasis accounts for the majority of cancer-related deaths, and one of the main pathways of spread is via the peripheral blood (PB). As such, circulating tumor cells (CTCs) may serve as an important indicator of disease progression and response to treatment.^1^^,^^2^ In clinical practice, conventional liquid biopsy methods for CTC enumeration involve drawing and analyzing fractionally small (7.5 mL) blood samples. However, these are known to be inaccurate (particularly for quantifying rare CTCs) and do not readily permit measurement of changes in CTC numbers over time.^3^^–^^5^ For pre-clinical studies in mice, the entire PB volume is typically drawn, which does not allow serial measurements in the same animal. To address these limitations, our lab developed “diffuse *in vivo* flow cytometry” (DiFC), which enables non-invasive, continuous monitoring of fluorescently labeled CTCs in mice.^6^^–^^9^ In the system, specially designed fiber-optic probes [Fig. 1(a)] are placed on the mouse skin approximately above the large blood vessels in the leg or tail [Figs. 1(b) and 1(c)]. Unlike microscopy-based *in vivo* flow cytometry approaches,^10^^–^^13^ DiFC uses highly scattered light that allows fluorescence detection of CTCs several millimeters into tissue. As CTCs pass through the instrument field of view (FOV), a probe detects transient fluorescent events, referred to as “peaks” [Fig. 1(d)]. We place two optical probes arranged 3 mm apart along the blood vessel. By requiring that signal peaks be sequentially detected by both probes, we can determine the direction of CTC flow and discriminate true CTC events from random motion or electronic artifacts that may also generate peak-like fluctuations in fluorescent signals (see Sec. 2.3).

Our previous DiFC signal processing approach only considered any fluorescent event exceeding a fixed amplitude threshold (five times the local background signal standard deviation, $\sigma_{\mathrm{bg}}$) as a CTC peak candidate (${\text{Peak}}_{\mathrm{cand}}$). True CTC peaks exhibit a characteristic waveform governed by the spatial sensitivity function of the DiFC probe, as shown by the Jacobian profile computed via Monte Carlo simulation in Fig. 1(e).^14^ The sensitivity profile as a function of time was derived assuming an average blood flow speed of 100 mm/s in mice.^6^ As such, reliance on a simple amplitude-based thresholding strategy neglects informative features encoded in the temporal shape of the signal. Because CTCs are exceedingly rare (as low as 1 CTC per mL of PB),^15^^,^^16^ false-positive signals are highly problematic. Mitigation of spurious detections from random signal noise required use of sufficiently high detection thresholds to minimize false positives, at the expense of potentially missing true, lower signal-to-noise ratio (SNR) peaks that fell below the threshold. Hence, an improved signal processing method that incorporates information contained in the signal shape is advantageous both for reducing false positives and improving true-positive counts.

To address this, we present a machine learning (ML)-integrated approach to more reliably distinguish true peaks from artifacts, particularly in noisy datasets.^17^ Convolutional neural networks (CNNs) are a class of ML models well suited for this task because they exhibit strong capability in extracting local features from time-series data^18^ and have been successfully applied in related signal-processing applications.^19^^–^^21^

## 2 Methods

### 2.1 Overview of the DiFC Signal Processing Problem

**Fig. 2:**
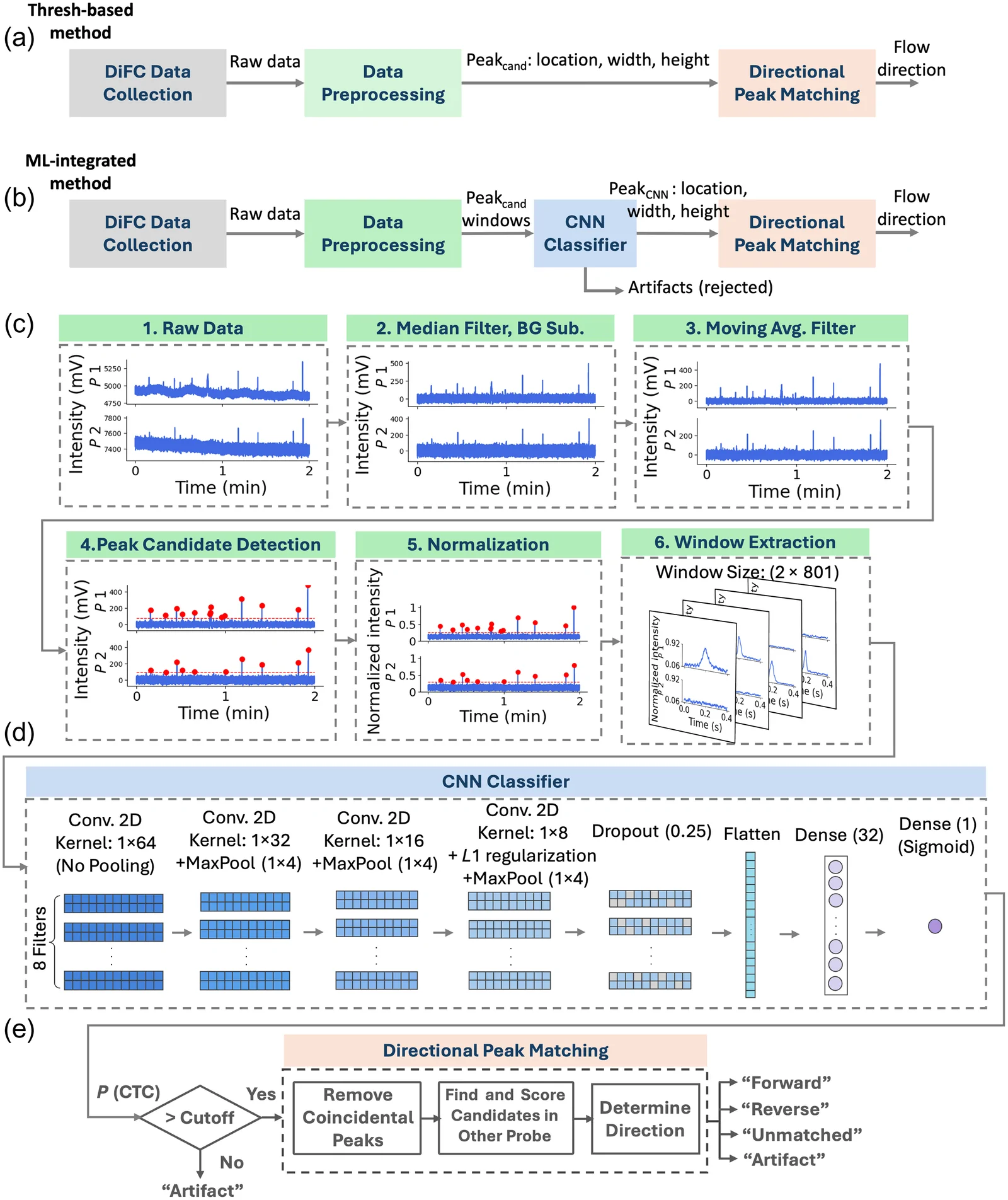
Overview of the signal processing workflow. (a) In the threshold-based approach, after pre-processing of raw data, all detected peak candidates (${\text{Peak}}_{\mathrm{cand}}$) were processed with the directional peak matching algorithm. (b) In the ML-integrated approach, peak candidates (${\text{Peak}}_{\mathrm{cand}}$) were first analyzed by a CNN classifier and only candidates classified as peaks (${\text{Peak}}_{\mathrm{CNN}}$) were processed by the directional peak matching algorithm. (c) Data pre-processing steps (BG Sub = background subtraction). (d) ML-based peak classification: After data collection and pre-processing, the data were passed through the CNN for the training phase, yielding the probability of each peak candidate being a true peak. (e) Directional peak matching model determined the direction of CTC movement.

The measured fluorescence comprised background tissue autofluorescence, transient peaks from fluorescently labeled CTCs, and false-positive signals arising from animal motion (e.g., breathing and twitching) and instrument electronic noise. The goal of the signal processing algorithm was to distinguish true CTC peaks from these background signals as accurately as possible. Figures 2(a) and 2(b) compare the previous threshold-based pipeline [Fig. 2(a)] with the ML-integrated pipeline introduced here [Fig. 2(b)]; the components of the latter are detailed in Secs. 2.2 and 2.3. In both pipelines, the input was the raw two-probe fluorescence time series, and the output was a set of detected events labeled as forward, reverse, unmatched, or artifact. Following pre-processing, peak candidates exceeding the detection threshold were identified independently on each probe, yielding a per-probe list of candidate locations, heights, and widths. In the threshold-based approach, these lists were passed directly to the directional matching algorithm. In the ML-integrated approach, the CNN classifier was first applied to each candidate to reject artifacts, and only the retained candidates were passed to the same directional matching algorithm.

### 2.2 Machine Learning Model

#### 2.2.1 Signal pre-processing and initial identification of peak candidates

Fluorescence data were acquired continuously from both optical fibers at a sampling rate of 2000 samples per second. We first applied a pre-processing algorithm to the raw data acquired from both optical fibers [Fig. 2(c)]. First, the background autofluorescence was removed from the signal by applying a sliding median filter with a window length of 2.5 s (5000 samples) and subtracting it from the raw signal. Next, a 3-ms (6 samples) smoothing operation was applied to the signal with a moving average filter. The peak candidates were then identified using the “find_peaks” function from the “scipy.signal” module in Python, defined as transient signals with amplitudes exceeding a fixed multiple of the standard deviation of the local signal ($4\sigma_{\mathrm{bg}}$). Peak detection was performed independently on each probe, so that each peak candidate corresponded to a detection on a single probe at a specific time; the temporal locations, widths, and heights of these candidates were recorded for further analysis. The data were then normalized to [0, 1] using the minimum and maximum values of each scan to facilitate uniform processing and prevent scale-related biases. Around each peak candidate, an 801-sample window (400 samples on each side of the candidate plus the center sample, corresponding to ∼0.4  s) was extracted from both probes over the same time interval, yielding a 2×801 input array (2 probes × 801 time samples) that served as a single input to the CNN classifier (see Sec. 2.2.2). The 801-sample (0.4 s) window length was chosen based on the distribution of peak candidate widths, which had a mean of 474 samples (≈0.24  s) and a median of 476 samples (≈0.24  s). The observed widths fell within the 801-sample window, so this size was chosen to contain the full temporal extent of a candidate peak while keeping the input compact.

#### 2.2.2 CNN classifier

The classifier comprised four two-dimensional convolutional layers, each with eight filters and a height 1 kernel (1×64, 1×32, 1×16, and 1×8). The kernel size was decreased across successive layers to extract temporal features at progressively finer scales. Because the kernel height was 1, each filter convolved along the time axis within an individual probe, with filter weights shared across the two probes; the two probe signals were not combined until the fully connected stage. The network depth was set empirically in relation to the available training data: a fourth convolutional layer was added to an initial three-layer network once the training set was enlarged, which supported the additional capacity. With the exception of the first layer, each convolutional layer was followed by a max pooling layer with a 1×4 kernel to reduce the temporal dimension. Max pooling was not applied after the first layer to avoid excessive downsampling, which would otherwise reduce the temporal dimension below the kernel length of a subsequent layer and yield invalid (negative) feature dimensions. An L1 kernel regularizer (λ=0.01) was applied to the fourth convolutional layer, and a dropout layer that randomly set 25% of inputs to zero during training was added after the convolutional stack to reduce overfitting.^22^^,^^23^ The output was then flattened and passed to two fully connected layers, a dense layer of 32 units followed by a single-unit output layer. The rectified linear unit activation was used for the convolutional layers and the first dense layer,^24^^,^^25^ while the final layer used a sigmoid activation to produce a value between 0 and 1, representing the probability that the input window contained a true peak from a fluorescently labeled or fluorescence-expressing cell. The CNN was trained on manually labeled windows (see Sec. 2.2.3) using the Adam optimizer and a binary cross-entropy loss. Training used a batch size of 4 over a maximum of 50 epochs, with the learning rate initialized at 0.001 and reduced by a factor of 10 every 10 epochs. These training hyperparameters were selected empirically by comparing configurations during 10-fold cross-validation and retaining those with the best validation accuracy. Early stopping was applied if the validation loss did not improve for three consecutive epochs, and the weights from the best-performing epoch were restored.^26^

The trained model was then applied to unseen DiFC data. Signal windows containing candidate peaks with predicted probabilities exceeding a predefined cutoff were classified as “1” (CNN-predicted peaks or ${\text{Peak}}_{\mathrm{CNN}}$) and passed to the directional matching algorithm for final classification, while all remaining windows were classified as “0” or artifacts. The probability cutoff was determined based on receiver operating characteristic (ROC) analysis and the distribution of predicted probabilities on the held-out validation set (see Sec. 3.1).

#### 2.2.3 Peak labeling and CNN training

The datasets used in this study were drawn from our previously published work as follows:

(i) Multiple myeloma (MM) disseminated xenograft model (DXM):^7^ Briefly, 8-week-old male SCID-bg mice were intravenously (i.v.) injected via the tail vein with MM.1S.GFP.Luc cells. After injection, MM cells rapidly homed to the bone marrow niche and proliferated, with CTCs beginning to disseminate into the bloodstream ∼3 weeks later. Two cohorts of mice were monitored for up to ∼5 weeks post-injection. A blue-green GFP-compatible DiFC system operating at 2000 Hz was used to scan the tail of the mice bi-weekly, with each scan lasting 45 min.
(ii) L1210A leukemia model:^8^^,^^9^ Briefly, female athymic nude mice received L1210A leukemia cells labeled with a near-infrared fluorescent contrast agent (OTL38^27^) at 6 to 8 weeks of age. L1210A cells were either labeled *in vitro* with OTL38 prior to i.v. injection, or L1210A cells and OTL38 were i.v. injected separately into the same mouse. The tail or leg was then scanned with a near-infrared-compatible DiFC system operating at 2000 Hz for 1 h.

A total of 18 scans from CTC-bearing mice were used for manual labeling of the training, validation, and test datasets. These comprised 11 scans from the MM DXM (obtained from 7 SCID-bg mice) and 7 scans from the L1210A model (obtained from 3 athymic nude mice). In addition, 12 DiFC scans were acquired from 5 control mice injected with PBS only, comprising 2 SCID-bg mice scanned using the GFP-compatible DiFC system^7^ and 3 athymic nude mice scanned using the near-infrared-compatible DiFC system.^8^^,^^9^ Individual scans lasted from 45 min to 1 h.

**Fig. 3:**
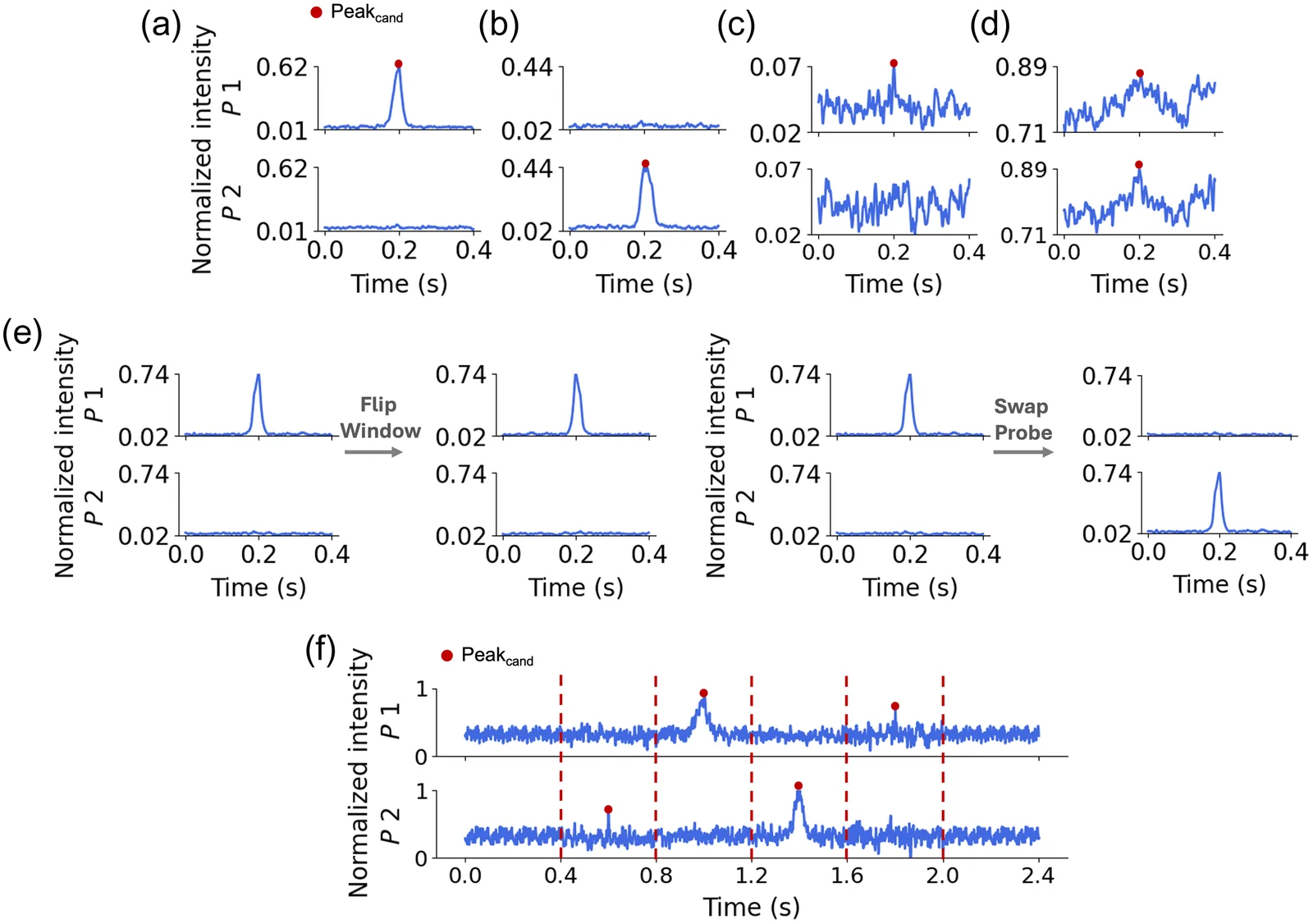
Example data windows, augmentation, and *in silico* data generation. Example 0.4-s data windows centered on peak candidates, showing a true peak from a fluorescently labeled cell detected by (a) probe 1 and (b) probe 2. False peak candidates may originate from (c) electrical noise and (d) motion artifacts. (e) Representative 0.4-s DiFC data window showing a true peak in probe 1, its flipped-window version, and a swapped-probe version. (f) Building block sequence used to generate the *in silico* dataset. From left to right: background noise, artifact in probe 2, true peak in probe 1, true peak in probe 2, artifact in probe 1, and background noise.

The peak candidates identified during pre-processing were manually inspected and labeled as “1” if attributed to a fluorescent cancer cell passing through the optical fiber and as “0” if attributed to artifacts such as electrical noise, motion, or breathing. Labeling was performed by trained readers using established morphological and temporal criteria to distinguish true peaks from artifacts. True peaks exhibited a characteristic bell-shaped profile with measurable width and distinct peak area [Figs. 3(a) and 3(b)], whereas electrical noise appeared as abrupt, sharp spikes [Fig. 3(c)]. In addition to peak shape, a matching peak detected in the signal from the other probe with an expected time delay provided further support for a true positive. Conversely, coincident peaks appearing simultaneously in the signals from probes 1 and 2 were considered artifacts [Fig. 3(d)]. Any peak candidates identified in scans performed on control mice were automatically labeled as artifacts since it was known *a priori* that no fluorescently labeled cells were present in circulation. In total, 2200 true-peak windows (“1”) and 4099 artifact windows (“0”) were annotated, where each window is a 2×801 array associated with a candidate detected on one probe, with candidates from probes 1 and 2 pooled. We partitioned the data at the scan level, so that all candidates from a given scan fell entirely within the training, validation or the test set, with both cell lines represented in each (11 training scans, 5 validation scans, and 14 test scans). To construct balanced training, validation, and test sets, artifact windows were randomly selected from the full artifact set within each split. This yielded 3054 training windows, 540 validation windows, and 806 test windows.

We balanced the classes by downsampling artifacts rather than applying a weighted loss because the balanced-dataset approach kept the predicted probabilities well-calibrated around the decision boundary—important here since the operating cutoff was selected directly from the predicted-probability distribution and ROC analysis on the validation set (see Sec. 3.1).

#### 2.2.4 Data augmentation

We used data augmentation to expand the training dataset and reduce overfitting.^28^ We applied two strategies: “flipped-window” and “swapped-probes.”^29^ In the flipped-window approach, each training example was reversed in time while retaining its original label. In the swapped-probes approach, the signals from probes 1 and 2 were exchanged, such that a true peak originally present in probe 1 appeared in probe 2 after swapping, and vice versa. Figure 3(e) shows a representative true peak and its flipped-window and swapped-probe version. Both data augmentation methods were applied to a randomly selected 25% subset of the training set, expanding the training set from 3054 to 4581 examples.

### 2.3 Directional Peak Matching

The directional peak matching algorithm was applied as previously described.^6^^,^^7^ First coincident peaks were labeled as “artifact” and excluded from further analysis. Then, for each peak candidate, a search interval on the other probe was defined using an estimated cell speed derived from peak width. Potential matches within this interval were identified, and the best match was selected based on similarity in peak width, height, and inferred speed. Each matched pair was labeled as “forward” or “reverse” based on the relative timing of the two peaks, and candidates without a suitable match were recorded as “unmatched.”

In the previous threshold-based approach, all peak candidates were passed to directional matching, and any coincident peaks were counted as artifacts. In the ML-integrated approach, only ${\text{Peak}}_{\mathrm{CNN}}$ candidates were passed to matching; the rest of the data processing pipeline remained unchanged. The artifact set in the ML-integrated method therefore comprises both the candidates rejected by the CNN and the coincident peaks removed during directional matching.

### 2.4 Evaluation Datasets

To evaluate the complete pipeline of the ML-integrated algorithm on unseen data and compare it with the threshold-based method, we used DiFC scans from *in silico* datasets, control mice, and CTC-bearing mice, as follows. These evaluation scans were distinct from, and did not overlap with, the scans used for the training, validation, and test datasets in Sec. 2.2.3.

#### 2.4.1 In silico data

Prior to testing our algorithm on experimental DiFC data, we generated an *in silico* dataset to evaluate model performance under fully known conditions. Because the exact number, type, direction, and timing of every event are prescribed by construction rather than measured, this dataset is the only one for which the directional output of the complete pipelines can be verified—which was not possible for CTC-bearing *in vivo* scans. We generated a library of three building block types: background noise, true peak, and artifact blocks, each extracted from real *in vivo* data that were excluded from all training, validation, test, and evaluation sets. All blocks were 0.4-s windows: true-peak and artifact blocks were centered on manually labeled peak candidates, while background noise blocks contained pre-processed autofluorescence signal with minimal fluctuations. Blocks were selected with near-zero endpoint values and small variation to ensure smooth stitching and were then concatenated to generate a 10-min *in silico* scan. We used a structured sequence of segments: background noise, artifact in probe 2, true peak in probe 1, true peak in probe 2, artifact in probe 1, and background noise again [Fig. 3(f)]. We previously observed that incorrect matches occurred when peaks and artifacts were detected within a narrow temporal window, producing false positives and incorrect matches. We therefore deliberately designed this sequence so that true peaks and artifacts co-occur across both probes, reproducing a known failure mode in which the threshold-based method produces incorrect directional matches. We hypothesized that reducing the detection of false positives would improve directional matching outcome.

#### 2.4.2 Control (non-CTC bearing) mice

Scans from control, non-CTC bearing mice were used to evaluate algorithm performance (since all detected peak candidates were, by definition, artifacts). We used a total of six scans lasting 45 min each: three acquired with the GFP-compatible DiFC system and three with the near-infrared-compatible DiFC system.

#### 2.4.3 CTC-bearing mice

We analyzed 360 min of data from seven scans acquired in CTC-bearing mice, four of which were from GFP-expressing MM-bearing mice and three from mice injected with labeled L1210A cells (models described in Sec. 2.2.3). Unlike for the *in silico* and control datasets, ground truth was not known *a priori*; therefore, each scan was manually reviewed and labeled.

### 2.5 Metrics

To evaluate candidate peak classification performance (${\text{Peak}}_{\mathrm{CNN}}$ versus artifacts) of the CNN classifier, we computed accuracy, precision, sensitivity, and specificity, as defined in Eqs. (1)–(4), using the test dataset described in Sec. 2.2.3:

$$\text{Accuracy}=\frac{\text{True Positives}+\text{True Negatives}}{\text{True Positives}+\text{True Negatives}+\text{False Positives}+\text{False Negatives}},$$ (1)

$$\text{Precision}=\frac{\text{True Positives}}{\text{True Positives}+\text{False Positives}},$$ (2)

$$\text{Sensitivity}=\frac{\text{True Positives}}{\text{True Positives}+\text{False Negatives}},$$ (3)

$$\text{Specificity}=\frac{\text{True Negatives}}{\text{True Negatives}+\text{False Positives}}.$$ (4)

We also calculated the area under the ROC curve (AUC), which illustrated the relationship between true-positive rate (TPR) and false-positive rate (FPR) across classification probability cutoff values. The TPR measured the proportion of true peaks correctly identified by the model and was equivalent to sensitivity, as defined in Eq. (3). The FPR quantified the proportion of artifacts incorrectly classified as true peaks and was computed as the ratio between the number of false positives and the total number of artifacts, defined in Eq. (5):

$$\text{False-Positive Rate }(\mathrm{FPR})=\frac{\text{False Positives}}{\text{False Positives}+\text{True Negatives}}.$$ (5)

To evaluate the directional peak matching algorithm on the evaluation datasets described in Sec. 2.4, we assessed whether matches were correctly or incorrectly classified by direction. Correct matches were true forward or reverse peak pairs correctly identified by the algorithm, whereas incorrect matches were artifact or unmatched peaks misclassified as forward or reverse. For clarity in the evaluation of the ML-integrated approach, key classification terms are defined in Table 1.

**Table 1:** Description of key classification terms used in ML-integrated DiFC signal processing. F, R, and U represent “forward,” “reverse,” and “unmatched,” respectively.

Term	Brief description
Peak (true peak)	Transient fluorescent signal caused by a CTC detected in the FOV
Artifact	Signal fluctuation from non-biological sources (e.g., noise and motion)
${\text{Peak}}_{\mathrm{cand}}$	Signal transient exceeding threshold
${\text{Peak}}_{\mathrm{CNN}}$	Peak candidate classified as a peak by the CNN
Forward	Peak pair indicating CTC movement from first to second probe
Reverse	Peak pair indicating CTC movement from second to first probe
Unmatched	True peak with no match on the alternate probe in the search interval
Missed	True peak below detection threshold, excluded as a peak candidate
True positive	True peak correctly classified by CNN or labeled F/R/U in directional matching
False positive	Artifact incorrectly accepted by CNN or labeled F/R/U in directional matching
True negative	Artifact correctly rejected by CNN or directional matching
False negative	True peak incorrectly rejected by CNN or directional matching
Correct match	Correctly identified forward or reverse peak pairs
Incorrect match	Artifacts or unmatched peaks incorrectly labeled as pairs

## 3 Results

### 3.1 Model Training and Characterization

**Fig. 4:**
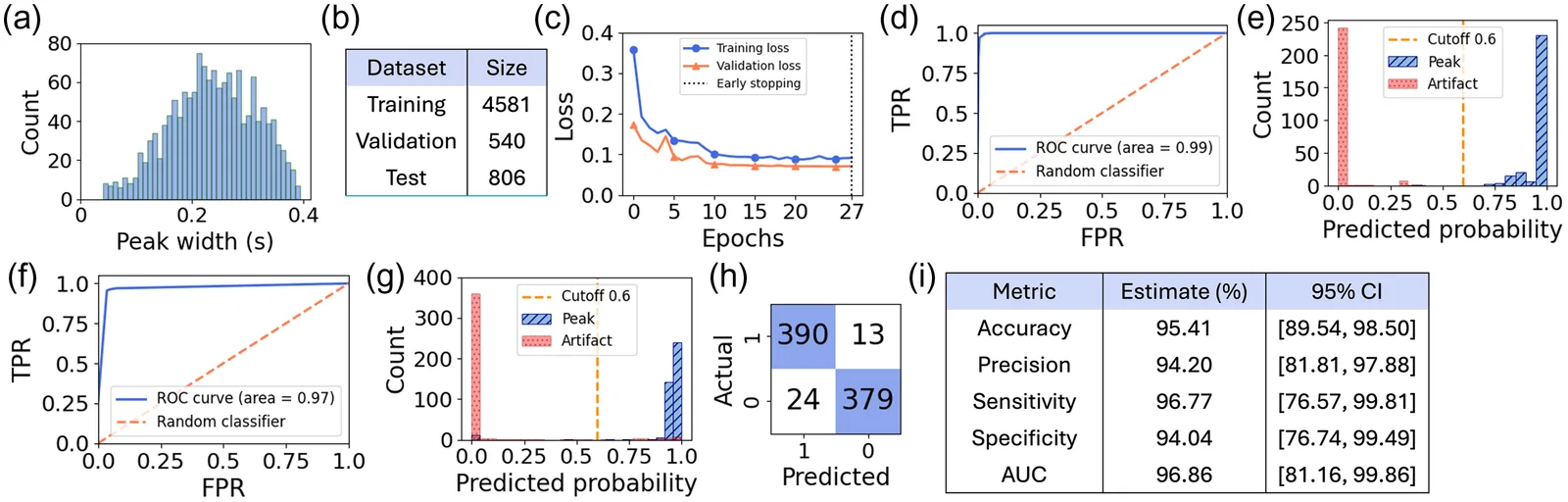
CNN classifier training and performance. (a) Distribution of peak candidate widths. (b) Training, validation, and test dataset sizes. (c) Training and validation loss over training epochs. Training stopped at the 27th epoch, when the validation loss did not improve for three consecutive epochs. (d) ROC curve for the CNN classifier on the validation dataset. (e) Histogram of the predicted probabilities for peak candidates in the validation set, with true peaks shown in blue and artifacts shown in red. Predicted probabilities for true peaks are close to 1, while those for artifacts are close to 0. The orange dashed line indicates the selected probability cutoff for the CNN classifier. (f) ROC curve for the CNN classifier in the test dataset. (g) Histogram of the predicted probabilities for peak candidates in the test set. (h) Peak classification confusion matrix using the CNN classifier on the test set with the cutoff set to 0.6. “0” and “1” represent artifact and peak, respectively. (i) Accuracy, precision, sensitivity, specificity, and AUC of the CNN classifier on the test dataset, with their corresponding 95% confidence intervals.

We first characterized the performance of the CNN classifier independent of directional matching. Figure 4(a) shows the distribution of peak candidate widths that motivated the 0.4 s classifier input window, and Fig. 4(b) summarizes the training (4581), validation (540), and test (806) set sizes. The binary cross-entropy loss over epochs for the training and validation sets is shown in Fig. 4(c). Early stopping was applied based on the validation loss with a patience of three epochs, restoring the model to its best weights before training stopped; here, training stopped at the 27th epoch. Because regularization and dropout were applied during training to improve generalization, the training loss was slightly higher than the validation loss. The CNN classifier was trained on an Apple M3 Pro processor with 18 GB of memory and required ∼31  min of total CPU time.

We next evaluated the trained CNN model on the validation dataset to select the probability cutoff. The ROC curve generated by varying the classifier probability cutoff in the range [0, 1] is shown in Fig. 4(d). The distribution of predicted probabilities for both true peaks and artifacts in the validation set was also analyzed [Fig. 4(e)]. As shown, the predicted probabilities for true peaks (blue) were close to 1, while those for artifacts (red) were near 0. By examining both the ROC curve and the predicted probability distribution, we selected 0.6 as a peak candidate cutoff that balanced low-false-positive and high-true-positive performance, although this can be adjusted to trade off the two considerations. This cutoff was then fixed and applied to the test set.

The ROC curve and the distribution of predicted probabilities in the test set are shown in Figs. 4(f) and 4(g). On the test set, the model’s accuracy, precision, sensitivity, specificity, and AUC all exceeded 94%, as shown in the corresponding confusion matrix and performance metrics [Figs. 4(h) and 4(i)]. Confidence intervals were computed using scan-level cluster bootstrap (2000 resamples, resampling unit = scan, probability cutoff = 0.6).

### 3.2 Performance on DiFC Datasets

#### 3.2.1 In silico data

**Fig. 5:**
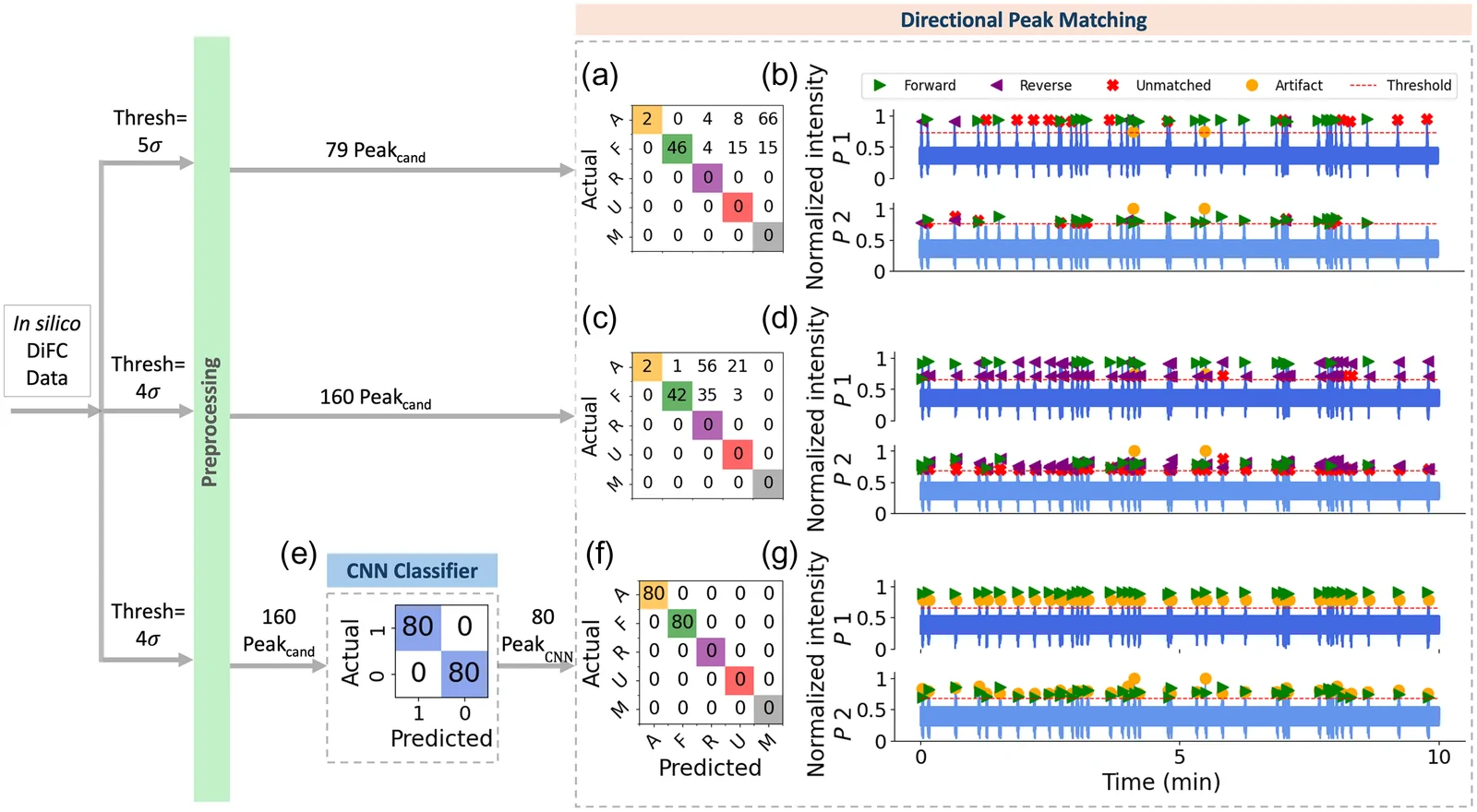
Performance comparison of the directional peak matching models on 10 min of *in silico* data. (a) Confusion matrix for the threshold-based directional peak matching with a threshold of $5\sigma_{\mathrm{bg}}$. “A,” “F,” “R,” “U,” and “M” are the abbreviated forms of artifact, forward, reverse, unmatched, and missed, respectively. (b) Color-coded predicted labels using the same model in panel (a). (c) Confusion matrix for the threshold-based directional peak matching with a threshold of $4\sigma_{\mathrm{bg}}$. (d) Color-coded predicted labels using the same model in panel (c). (e) Confusion matrix for the CNN classification on peak candidates detected with the threshold of $4\sigma_{\mathrm{bg}}$. “0” and “1” represent artifact and peak, respectively. (f) Confusion matrix for the ML-integrated directional peak matching with a threshold of $4\sigma_{\mathrm{bg}}$. (g) Color-coded predicted labels using the same model in panel (f).

In general, the ground truth number and direction of true peaks are not known for DiFC datasets measured in CTC-bearing mice *in vivo*. To evaluate our model, we first synthetically generated a 10-min scan containing 80 artifacts and 80 true peaks (all of which were in the forward direction) as described in Sec. 2.4.1. The results are summarized in Fig. 5, where the first and middle rows illustrate the result from our previously documented threshold-based approach^6^^,^^7^ with the threshold of $5\sigma_{\mathrm{bg}}$, and $4\sigma_{\mathrm{bg}}$, respectively. The last row corresponds to the results from the ML-integrated approach with the threshold of $4\sigma_{\mathrm{bg}}$.

The threshold-based approach with a threshold of $5\sigma_{\mathrm{bg}}$ identified 79 peak candidates for further analysis with the directional peak matching algorithm, whereas a further 81 lower amplitude peaks were missed as shown in the last column of the extended confusion matrix in Fig. 5(a). The higher threshold also reduced false positives by avoiding dimmer artifacts, as demonstrated in Fig. 5(a).

Lowering the threshold to $4\sigma_{\mathrm{bg}}$ [Figs. 5(c)–5(d)] captured more peak candidates, including both the lower-amplitude true peaks missed at $5\sigma_{\mathrm{bg}}$ and lower-amplitude artifacts. Because these artifacts were passed to the directional matching algorithm, many were paired with true peaks, producing an inflated number of reverse and otherwise incorrect matches. As a result, the number of correctly categorized forward-direction peaks did not increase but instead decreased relative to $5\sigma_{\mathrm{bg}}$ [from 46 to 42, Figs. 5(a) and 5(c)], while false positives rose to 78, of which 56 were mislabeled as reverse, 1 was mislabeled as forward, and the remaining 21 were mislabeled as unmatched.

Thus, rather than improving detection, lowering the threshold degraded the performance of the matching algorithm in the presence of abundant artifacts: the influx of artifact candidates not only generated incorrect matches but also displaced correct forward matches, reducing both true positives and correct matches compared with the higher threshold.

Next, with the lower threshold of $4\sigma_{\mathrm{bg}}$, we applied our CNN classifier prior to the peak matching algorithm (“ML-integrated approach”). As illustrated in the peak classification confusion matrix [Fig. 5(e)], the CNN classifier correctly classified all true peaks and artifacts. Only peak candidates classified as “1” (${\text{Peak}}_{\mathrm{CNN}}$) were passed to the directional peak matching step, removing artifacts from further analysis in the pipeline.

Following the directional matching step, all ${\text{Peak}}_{\mathrm{CNN}}$ were correctly labeled as forward, as shown in Fig. 5(f). Thus, the ML-integrated approach not only increased true positives and correct matches but also maintained a low number of false positives and incorrect matches.

#### 3.2.2 Control (non-CTC bearing) mice

We next applied our ML-integrated approach on DiFC datasets measured from non-CTC bearing mice, i.e., mice where it is known *a priori* that there are no true-positive peaks.

**Fig. 6:**
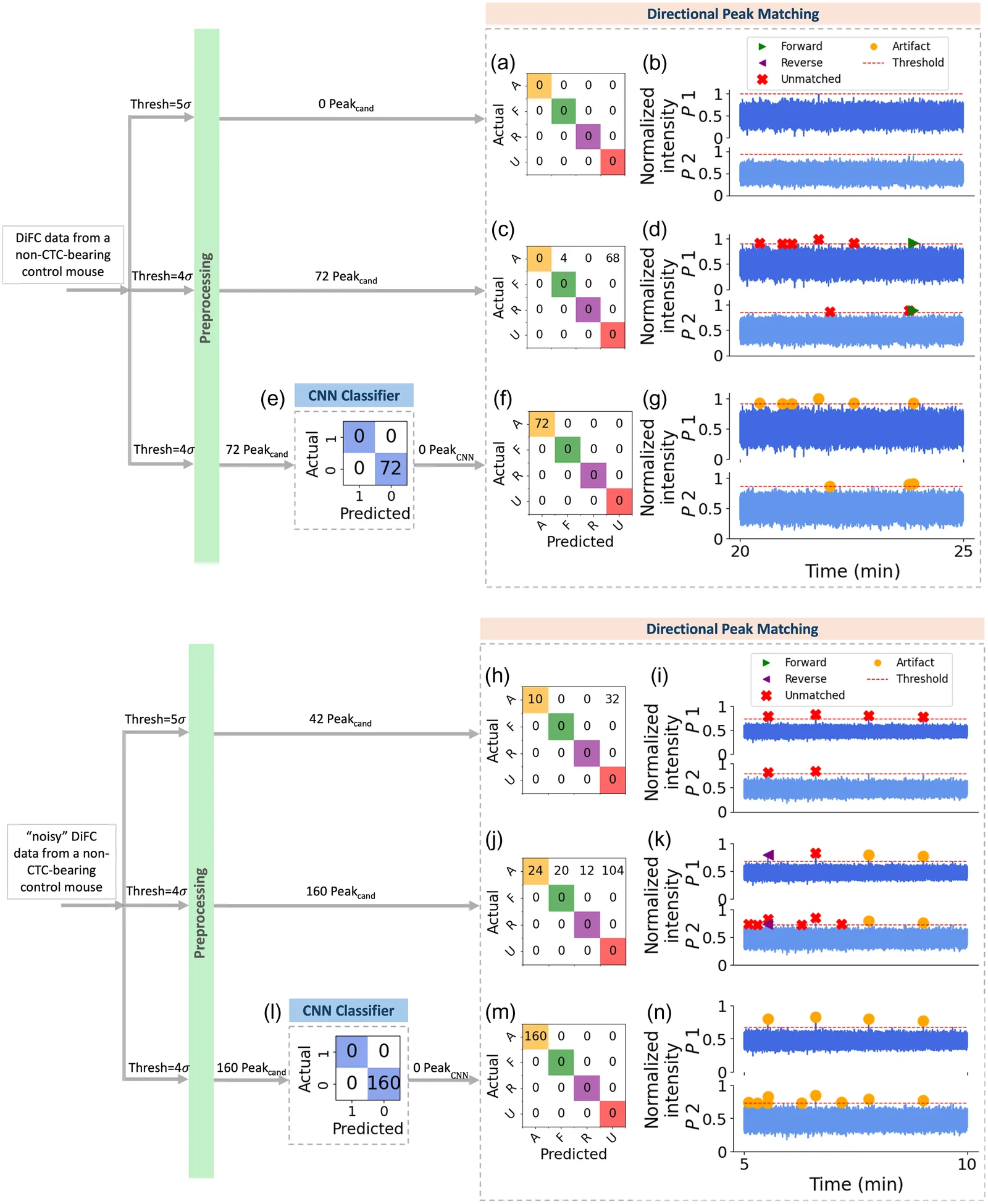
Performance on control (non-CTC bearing) mouse scans. Comparison of the threshold-based and ML-integrated directional peak matching on two control scans. (a)–(g) Representative scan. (h)–(n) Particularly noisy scan. Each scan is shown using the same panel layout. (a) and (h) Confusion matrix for threshold-based directional peak matching at $5\sigma_{\mathrm{bg}}$. (b) and (i) Color-coded predicted labels for the model in panels (a) and (h). (c) and (j) Confusion matrix for threshold-based directional peak matching at $4\sigma_{\mathrm{bg}}$. (d) and (k) Color-coded predicted labels for the model in panels (c) and (j). (e) and (l) Confusion matrix for the CNN classification on peak candidates detected at $4\sigma_{\mathrm{bg}}$. (f) and (m) Confusion matrix for ML-integrated directional peak matching at $4\sigma_{\mathrm{bg}}$. (g) and (n) Color-coded predicted labels for the model in panels (f) and (m).

Analysis of a typical dataset is shown in Figs. 6(a)–6(g). As shown in Fig. 6(a), application of our previously reported threshold-based method with a $5\sigma_{\mathrm{bg}}$ threshold resulted in detection of no peak candidates (and hence no false-positive detections). However, when the threshold was lowered to $4\sigma_{\mathrm{bg}}$ [Fig. 6(c)], 72 artifacts were identified as peak candidates, 4 of which were misclassified as forward and 68 as unmatched by the directional matching algorithm.

With the application of the CNN classifier after pre-processing [Fig. 6(e)], all 72 artifacts captured as peak candidates were classified as “0” (artifact), and none proceeded to directional matching, resulting in no false positives [Fig. 6(f)].

We analyzed a particularly noisy control dataset to evaluate the performance of the CNN and directional matching algorithm [Figs. 6(h)–6(n)]. Application of the threshold-based approach with a threshold of $5\sigma_{\mathrm{bg}}$ resulted in detection of 42 peak candidates, 32 of which were classified as unmatched by the directional matching algorithm and 10 as artifact through coincident peak removal.

When the threshold was lowered to $4\sigma_{\mathrm{bg}}$, 160 peak candidates were detected, of which 128 were classified as unmatched or coincident peaks. Of the remaining peaks, 20 and 12 were erroneously matched in forward and reverse directions, respectively [Fig. 6(j)]. In contrast, using the same threshold of $4\sigma_{\mathrm{bg}}$, the CNN model accurately classified all peak candidates as “0” (artifacts), resulting in no erroneous directional peak matches [as shown in Figs. 6(l)–6(m)]. This demonstrated the robustness of the ML-integrated approach in rejecting artifacts even under noisy conditions.

**Fig. 7:**
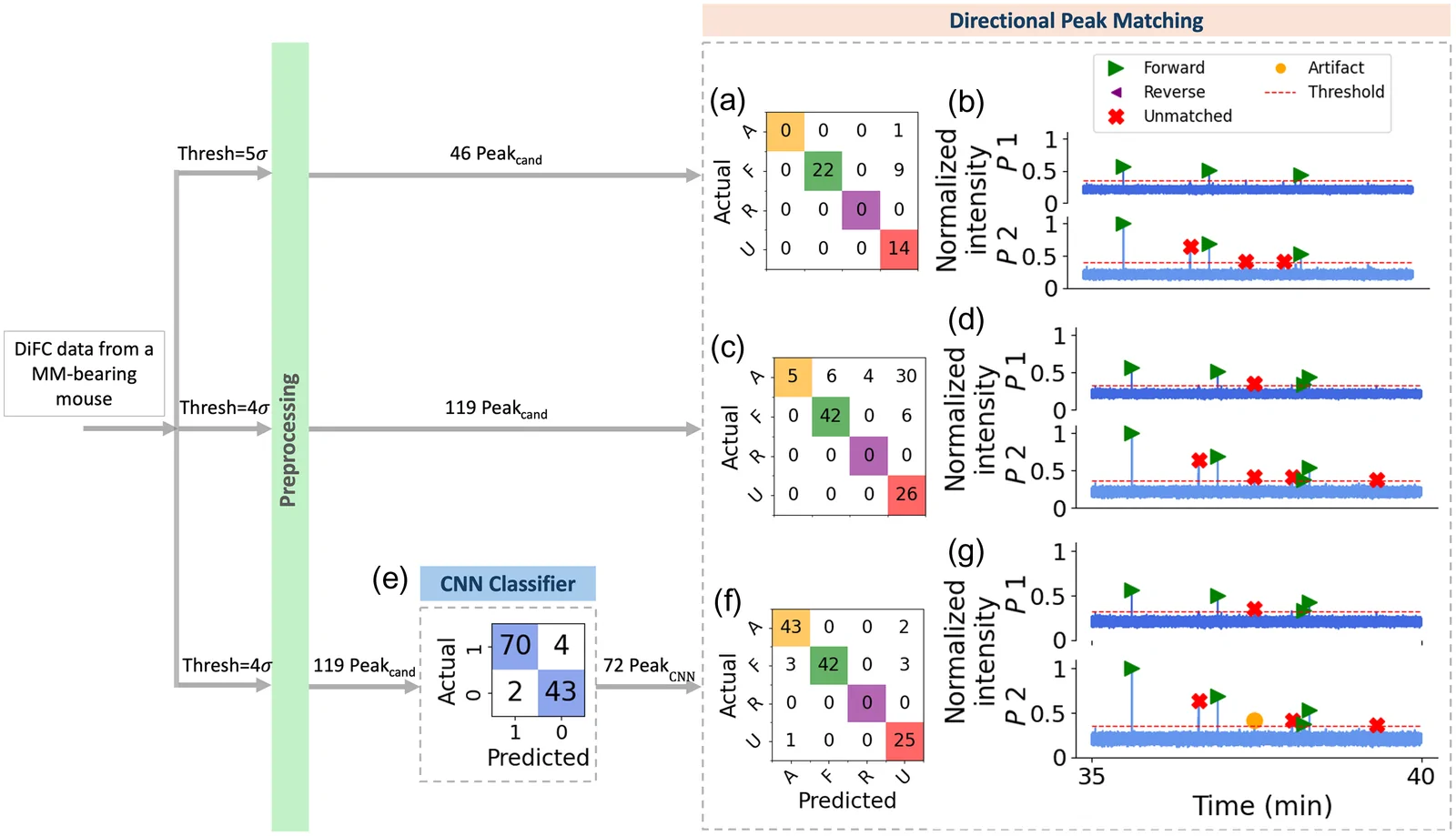
Performance comparison of the directional peak matching models on 45 min of data collected from an MM-bearing mouse. (a) Confusion matrix for the threshold-based directional peak matching with a threshold of $5\sigma_{\mathrm{bg}}$. (b) Color-coded predicted labels using the same model in panel (a). (c) Confusion matrix for the threshold-based directional peak matching with a threshold of $4\sigma_{\mathrm{bg}}$. (d) Color-coded predicted labels using the same model in panel (c). (e) Confusion matrix for the CNN classification on peak candidates detected with the threshold of $4\sigma_{\mathrm{bg}}$. (f) Confusion matrix for the ML-integrated directional peak matching with a threshold of $4\sigma_{\mathrm{bg}}$. (g) Color-coded predicted labels using the same model in panel (f).

**Fig. 8:**
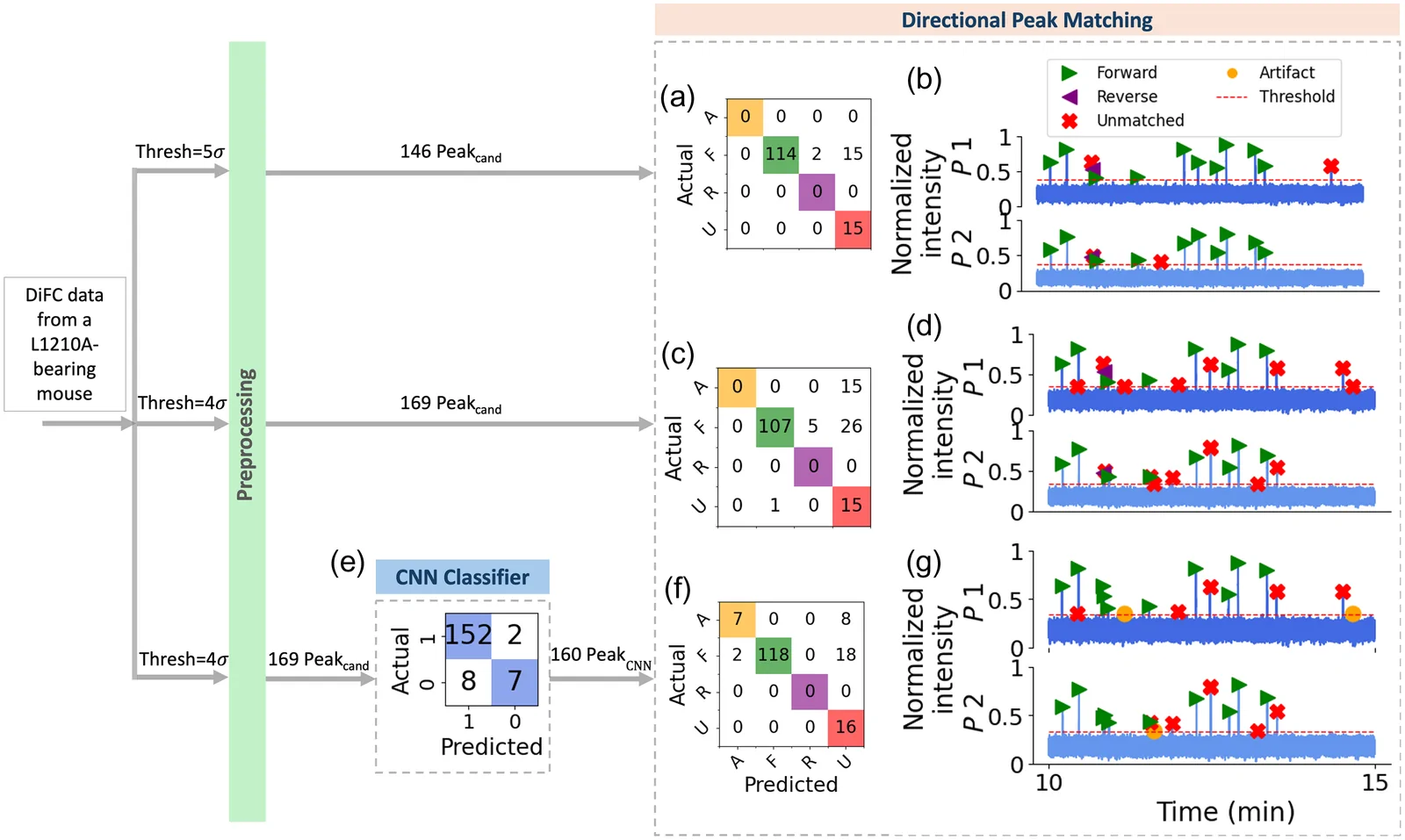
Performance comparison of the directional peak matching models on 45 min of data collected from an L1210A-bearing mouse. (a) Confusion matrix for the threshold-based directional peak matching with a threshold of $5\sigma_{\mathrm{bg}}$. (b) Color-coded predicted labels using the same model in panel (a). (c) Confusion matrix for the threshold-based directional peak matching with a threshold of $4\sigma_{\mathrm{bg}}$. (d) Color-coded predicted labels using the same model in panel (c). (e) Confusion matrix for the CNN classification on peak candidates detected with the threshold of $4\sigma_{\mathrm{bg}}$. (f) Confusion matrix for the ML-integrated directional peak matching with a threshold of $4\sigma_{\mathrm{bg}}$. (g) Color-coded predicted labels using the same model in panel (f).

We note that for the *in vivo* scans (Figs. 6–8), the color-coded label panels display a representative 5-min interval of each scan to more clearly show the behavior of each method; the corresponding full-scan plots are provided in the Supplementary Material.

#### 3.2.3 CTC-bearing mice

Analysis of a DiFC scan measured from an MM-DXM bearing mouse is summarized in Fig. 7. Application of the threshold-based method with a threshold of $5\sigma_{\mathrm{bg}}$ resulted in detection of 46 peak candidates, of which only 22 were correctly forward-matched and 14 were correctly unmatched peaks. Lowering the threshold to $4\sigma_{\mathrm{bg}}$ increased the number of detected candidates to 119; using the threshold-based method, this yielded 42 correct forward matches and 26 correct unmatched peaks, however, at the cost of substantially increased false positives (40 artifacts labeled as forward, reverse, or unmatched) and incorrect matches (6 artifacts matched as forward and 4 as reverse) after directional peak matching [Fig. 7(c)]. Using the ML-integrated approach with a threshold of $4\sigma_{\mathrm{bg}}$, the CNN classifier identified 72 of the 119 detected peak candidates as ${\text{Peak}}_{\mathrm{CNN}}$ (70 true peaks and 2 artifacts) and rejected the remaining 47 as artifacts [43 true artifacts and 4 true peaks; Fig. 7(e)]. Subsequent directional matching of the 72 ${\text{Peak}}_{\mathrm{CNN}}$ yielded 42 correct forward matches and 25 correct unmatched peaks, with no incorrect matches [Fig. 7(f)].

These results highlight the ML-integrated model’s ability to address the limitations of threshold-based methods across varying thresholds.

We next analyzed data in the same manner from L1210A-bearing mice. Lowering the threshold from $5\sigma_{\mathrm{bg}}$ to $4\sigma_{\mathrm{bg}}$ in the threshold-based approach increased the number of peak candidates from 146 to 169, and consequently increased the number of false positives from 0 to 15 (15 artifacts labeled as unmatched), as shown in Figs. 8(a) and 8(c).

In the ML-integrated approach with a threshold of $4\sigma_{\mathrm{bg}}$, the 169 peak candidates were first processed by the CNN classifier, which filtered out 9 candidates (represented in the “0” column of the 2×2 confusion matrix in Fig. 8(e), including 7 actual artifacts). The remaining 160 peak candidates were then passed to the directional peak matching algorithm. This reduced false positives from 15 to 8 and increased the number of correct forward matches from 107 to 118 compared with the threshold-based method, with no incorrect matches after directional peak matching [Fig. 8(f)].

We note that the extended confusion matrices shown in the output of directional peak matching for the *in vivo* evaluation datasets (Figs. 6–8) do not include a ”missed” row or column, unlike the matrices for *in silico* scans in Fig. 5, i.e., because the exact number of true peaks was not known *a priori*.

#### 3.2.4 Overall results

**Fig. 9:**
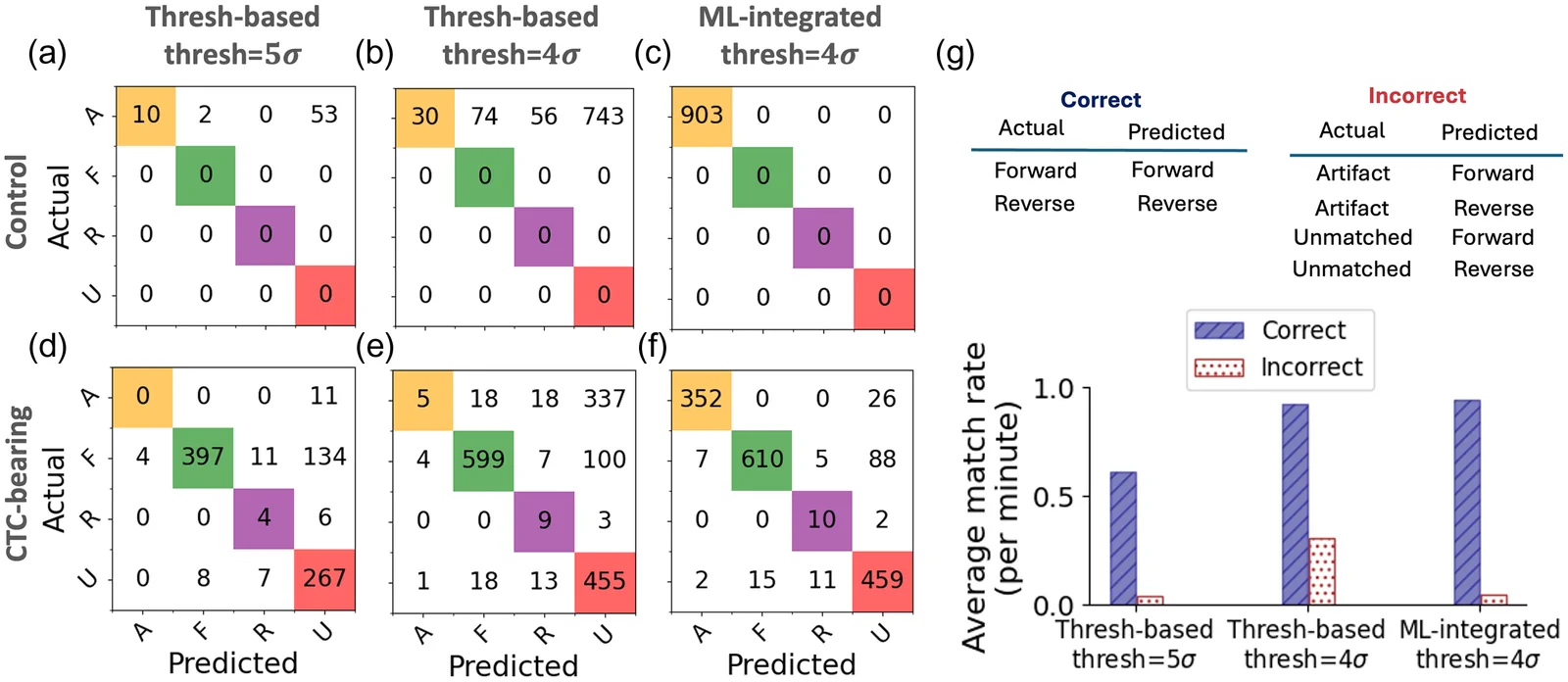
Performance of threshold-based and ML-integrated directional peak matching, derived from manually labeled evaluation scans collected from control and CTC-bearing mice. (a) and (b) Confusion matrices for threshold-based directional peak matching on control data at $5\sigma_{\mathrm{bg}}$ and $4\sigma_{\mathrm{bg}}$. (c) Confusion matrix for ML-integrated directional peak matching on control data at $4\sigma_{\mathrm{bg}}$. (d) and (e) Confusion matrices for threshold-based directional peak matching on CTC-bearing data at $5\sigma_{\mathrm{bg}}$ and $4\sigma_{\mathrm{bg}}$. (f) Confusion matrix for ML-integrated directional peak matching on CTC-bearing data at $4\sigma_{\mathrm{bg}}$. (g) Bar plot of correct versus incorrect match rate. Correct matches are defined as forward or reverse peaks correctly labeled. Incorrect matches are defined as artifact or unmatched peaks incorrectly labeled as forward or reverse.

Figure 9 summarizes the performance of the algorithm across all control and CTC-bearing evaluation data (Secs. 2.4.2 and 2.4.3) for the threshold-based and ML-integrated methods. As shown in Figs. 9(a)–9(c), lowering the threshold in the threshold-based method increased the number of false positives from 55 to 873 whereas the ML-integrated approach produced no false positives, with all 903 artifacts in control scans correctly classified. For the CTC-bearing scans, although lowering the threshold to $4\sigma_{\mathrm{bg}}$ in the threshold-based approach increased the number of correct matches [from 397 correct forward and 4 correct reverse peaks to 599 correct forward and 9 correct reverse peaks; Figs. 9(d) and 9(e)], it also increased false positives (from 11 to 373) and incorrect matches (from 15 to 67). The ML-integrated approach at the same threshold not only yielded a comparable number of correct matches (610 correct forward and 10 correct reverse peaks) but also maintained substantially fewer false positives (26) and incorrect matches (26), as illustrated in Fig. 9(f).

Last, Fig. 9(g) shows the correct versus incorrect matches for the threshold-based approach with thresholds of $5\sigma_{\mathrm{bg}}$ and $4\sigma_{\mathrm{bg}}$, and for the ML-integrated approach with a threshold of $4\sigma_{\mathrm{bg}}$. These results are based on all evaluation scans from both control and CTC-bearing mice, as described in Secs. 2.4.2 and 2.4.3. The average rate was computed as the total number of correct (or incorrect) matches summed across all evaluation scans, divided by the total scan duration in minutes. As shown, the ML-integrated approach achieved a high number of correct matches (comparable to the threshold-based approach at $4\sigma_{\mathrm{bg}}$) while maintaining a low number of incorrect matches (comparable to the threshold-based approach at $5\sigma_{\mathrm{bg}}$), effectively addressing the limitations of the threshold-based models.

It is also worth noting that applying the complete trained ML-integrated pipeline (pre-processing, CNN classification, and directional peak matching) to a single full-length DiFC scan (45 min) required ∼7.8  s of wall-clock time on a laptop CPU without GPU acceleration; one-time model and data loading added less than 1 s combined.

## 4 Discussion and Conclusion

In this study, we developed and validated a CNN classifier for peak detection in DiFC. By incorporating scans from two cancer cell lines, two mouse strains, and both our blue-green (GFP–compatible) and near-infrared-compatible DiFC systems, we evaluated the model across a range of fluorophores, signal characteristics, and instrument configurations and observed consistent performance across these conditions. This robustness across the experimental settings tested highlights the classifier’s potential for broader DiFC applications.

The results consistently showed that the CNN classifier addresses key limitations of our previously reported threshold-based methods. Specifically, when using a high threshold, threshold-based approaches reduce false positives but fail to capture lower SNR peaks, leading to missed true positives. Conversely, lowering the threshold captures more true positives but significantly increases false positives, especially in noisy datasets. The CNN classifier, however, incorporates both the shape and the amplitude of peak candidates, thereby achieving a balance by capturing a higher number of true positives while maintaining a low rate of false positives.

The advantages of the CNN classifier become particularly evident in scenarios with noisy data, where artifacts and fluctuations in the signal are prevalent. Threshold-based methods struggled in such conditions, often capturing artifacts as peak candidates and producing numerous false positives. In contrast, the CNN classifier effectively distinguishes between true peaks and artifacts, demonstrating its superior performance in challenging environments. This shortcoming of the threshold-based approach arises in part because the threshold-based approach removes artifacts only when coincident peaks are detected in both probes. Under noisy conditions, a fluctuation present in both probes may exceed the detection threshold in one probe but not the other; the detected peak candidate then has no coincident counterpart and can be retained as unmatched, leading to a false positive. Because the CNN classifies each candidate based on its temporal shape and amplitude, it can reject such artifacts regardless of whether a sub-threshold counterpart is present in the other probe.

Despite the advantages, one limitation of this study is the absence of an independent ground truth for datasets from CTC-bearing mice. As we noted above, for control (non-CTC bearing) mice, all detected events are known *a priori* to be artifacts (since no CTCs are present), which provides an annotation-independent ground truth. Likewise, for the *in silico* datasets, all event labels are known by construction. As such, these two cases provide independent ground truth to evaluate performance of our analysis pipeline. For the CTC-bearing datasets, the *in vivo* metrics reflect agreement with manual annotation by experts rather than verified true-cell detection.

In addition, although other architectures could be applied to this task, we chose a CNN for its suitability to the localized temporal shape of DiFC peaks and its demonstrated success on analogous time-series problems;^18^^–^^21^ a systematic architecture comparison was beyond the present scope and is left to future work.

We also plan to deploy this approach in DiFC scans taken from awake, freely moving mice.^30^ In these scenarios, motion artifacts introduce significant challenges to the accuracy of signal interpretation. Threshold-based methods are prone to failure in such conditions, producing excessive false positives and compromising their reliability. The CNN classifier, with its ability to handle complex, noisy datasets, is well-suited to address these challenges, reinforcing its importance in the ongoing development of DiFC for dynamic and real-world experimental settings.

Likewise, this ML-enhanced approach to time-series signal processing may be useful for *in vivo* flow-cytometry-based approaches using intravital microscopy or photoacoustic designs.^3^^,^^5^^,^^31^^,^^32^

## Supplementary Material

10.1117/1.BIOS.3.3.032903.s01

## Data Availability

The datasets analyzed during this work and the Python code are available through the Synapse repository (DOI: 10.7303/SYN73915900).
