# Supplementary material for: Machine learning approach for enumeration of circulating cells with diffuse in vivo flow cytometry
Source: Biophotonics Discov. 2026 Sep 4;3(3):032903. doi: 10.1117/1.BIOS.3.3.032903 (PMC13557453; doi:10.1117/1.BIOS.3.3.032903)
Supplement: Supplementary file 1 [file BIOS_003_032903_SD001.pdf]

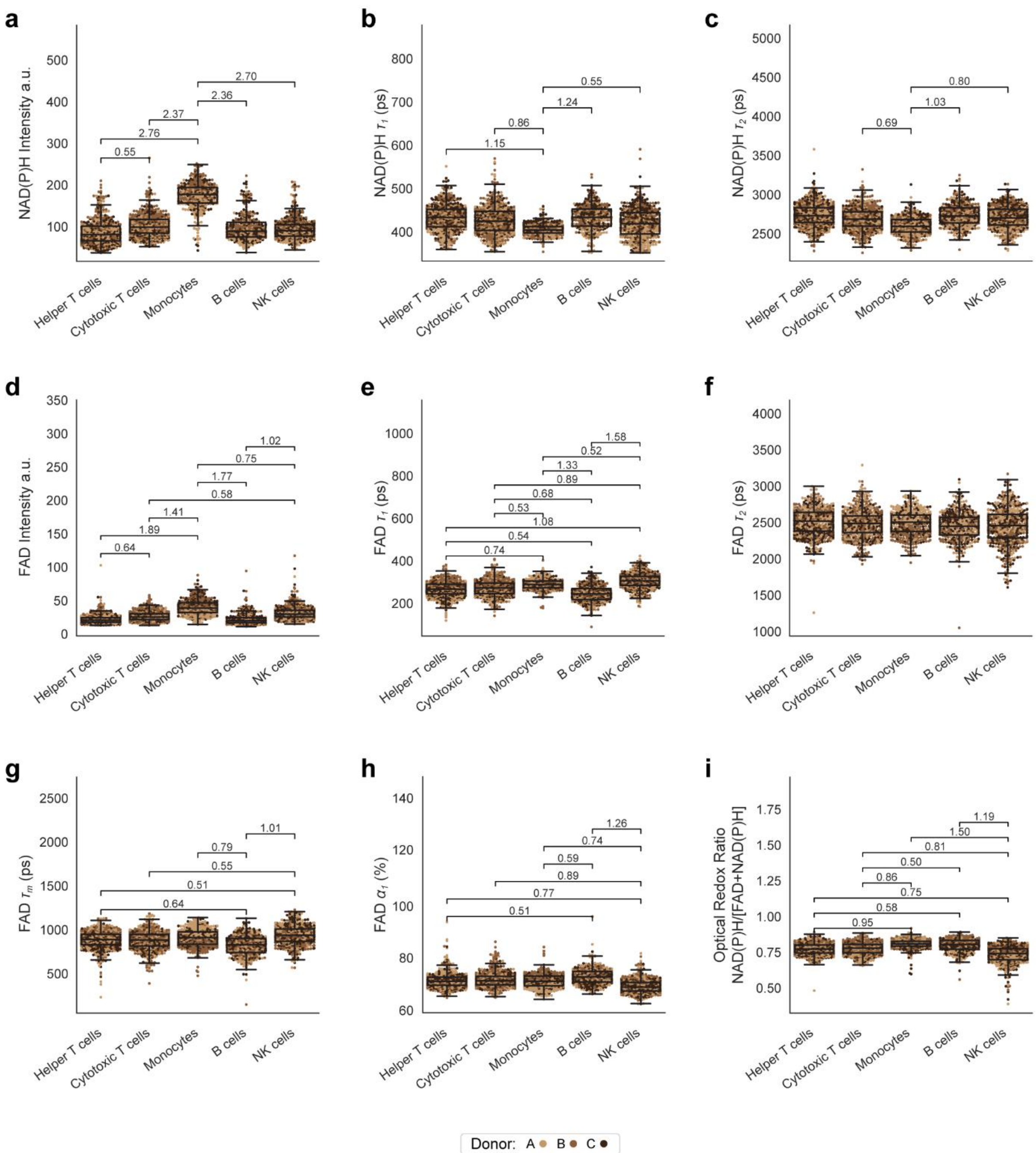

Supplemental Figure 1. OMI features of quiescent PBMC immune populations. (a-c) NAD(P)H features plotted by cell type. (d-h) FAD features plotted by cell type. (i) Optical redox ratio plotted by cell type. Data are displayed as box-and-whisker plots, representing the median and interquartile range (IQR), with whiskers at 1.5*IQR. Plots are overlaid on individually plotted cells shaded by donor, A, B, or C. n = 553 helper T cells, 554 cytotoxic T cells, 425 monocytes, 368 B cells, 510 NK cells. Effect size given as absolute value of Cohen's d. Insignificant and small effect sizes ($d<0.5$) not shown, medium ($0.5 \le |d| < 0.8$) and large ($|d| \ge 0.8$) effect sizes are shown. All comparisons with Cohen's d noted have $p<0.05$ with a Kruskal-Wallis test.

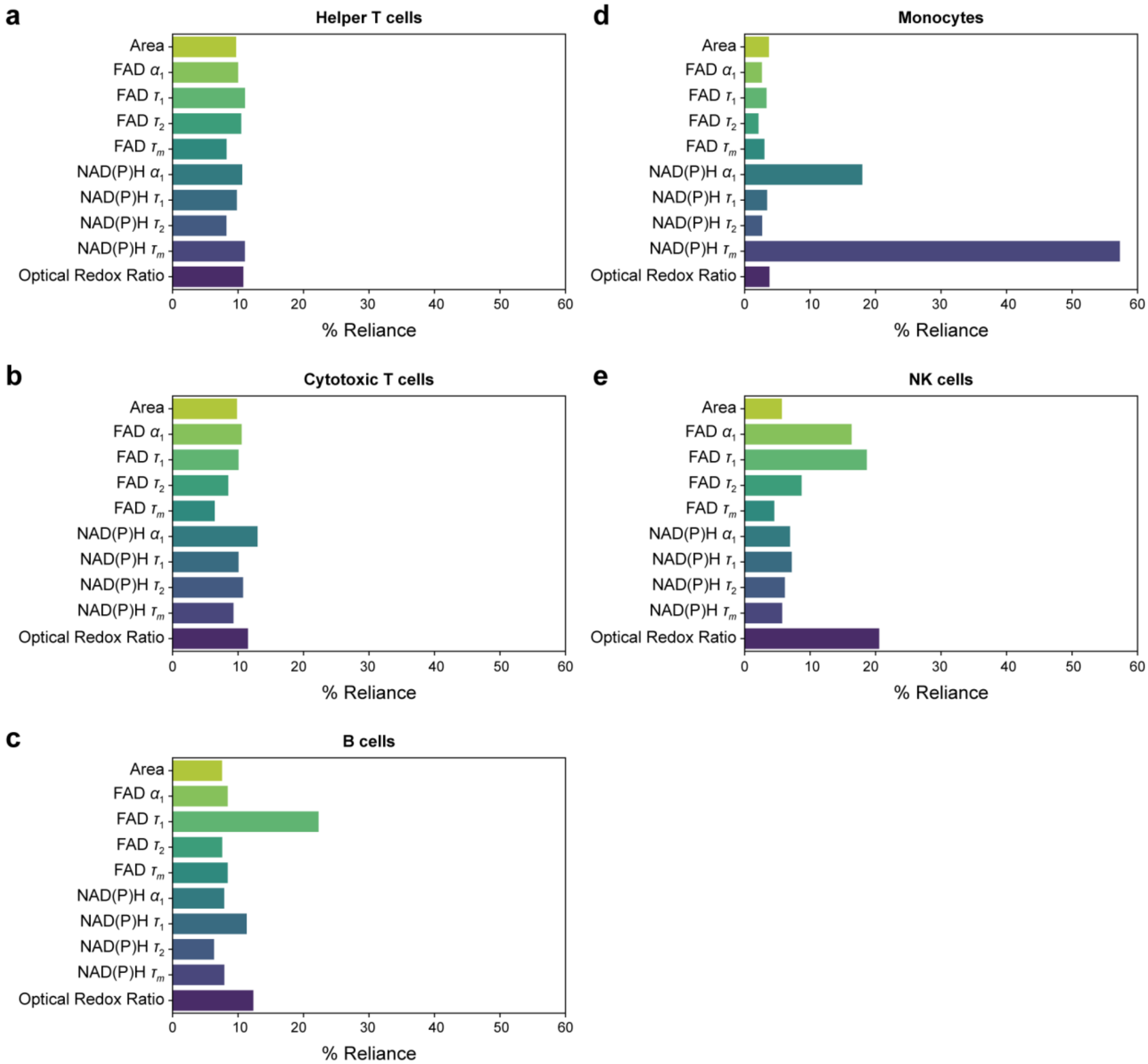

Supplemental Figure 2. Quiescent PBMC one versus rest random forest classification feature weights. Relative importance of the 10 OMI variables for classification of (a) helper T cells, (b) cytotoxic T cells, (c) B cells, (d) monocytes, and (e) NK cells from the rest of the PBMC immune populations.

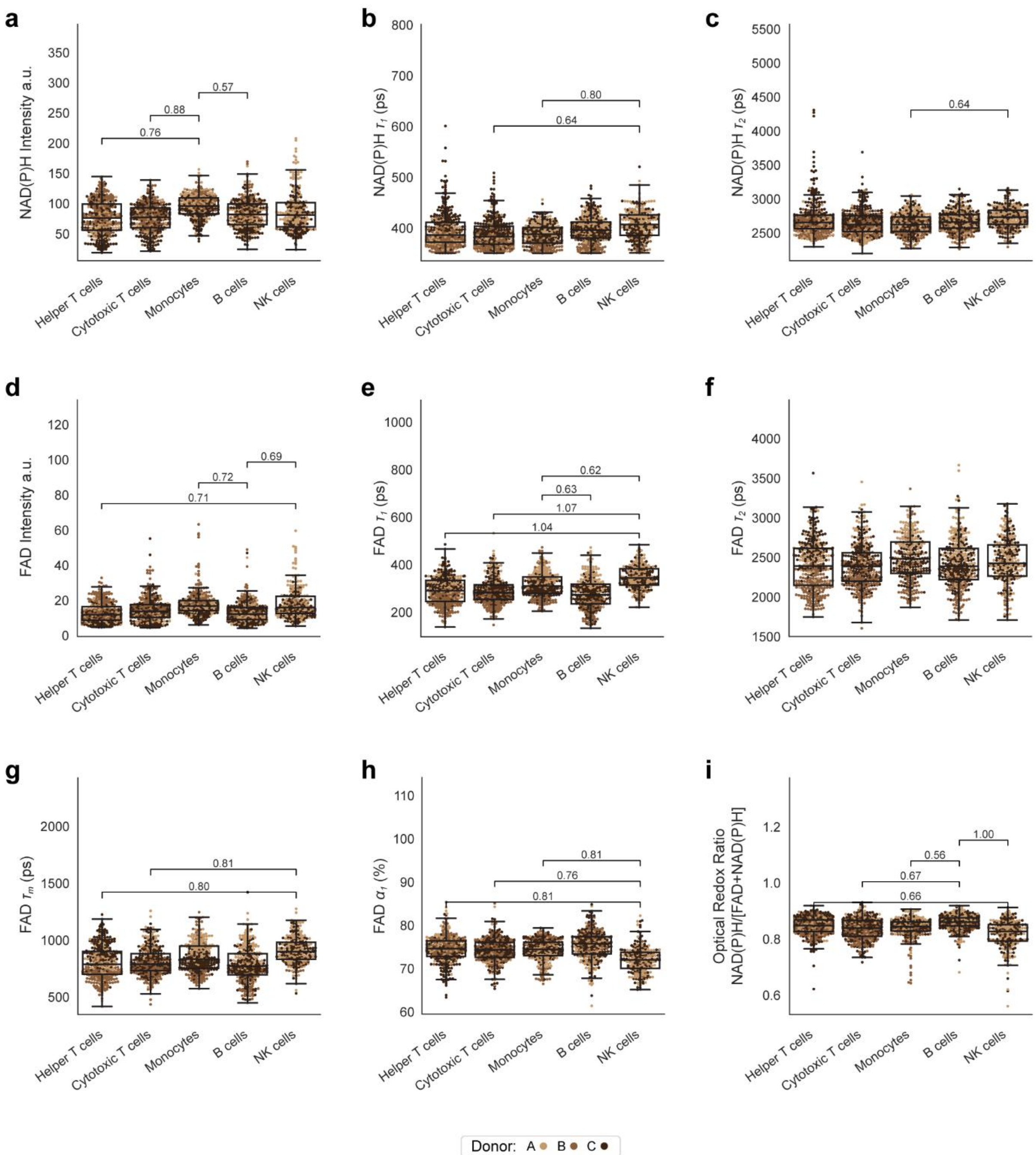

Supplemental Figure 3. OMI features of PMA and Ionomycin-activated PBMC immune populations. (a-c) NAD(P)H features plotted by cell type. (d-h) FAD features plotted by cell type. (i) Optical redox ratio plotted by cell type. Data are displayed as box-and-whisker plots, representing the median and interquartile range (IQR), with whiskers at 1.5*IQR. Plots are overlaid on individually plotted cells shaded by donor, A, B, or C. n = 363 helper T cells, 353 cytotoxic T cells, 305 monocytes, 340 B cells, 267 NK cells. Effect size given as absolute value of Cohen's d. Insignificant and small effect sizes (d<0.5) not shown, medium (0.5≤|d|<0.8) and large (|d|≥0.8) effect sizes are shown. All comparisons with Cohen's d noted have p<0.05 with a Kruskal-Wallis test.

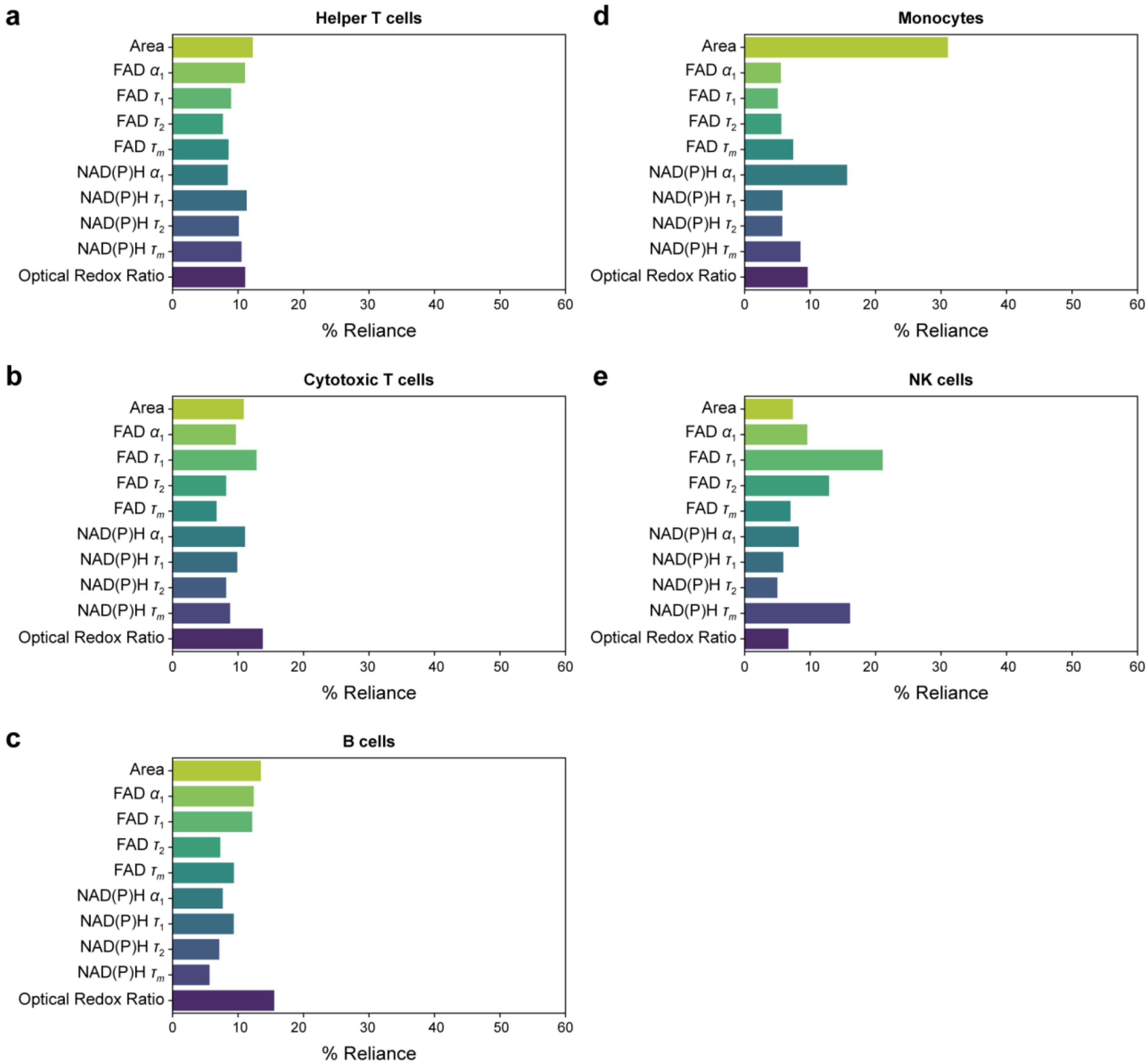

Supplemental Figure 4. PMA and Ionomycin-activated PBMC one versus rest random forest classification feature weights. Relative importance of the 10 OMI variables for classification of (a) helper T cells, (b) cytotoxic T cells, (c) B cells, (d) monocytes, and (e) NK cells from the rest of the PBMC immune populations.

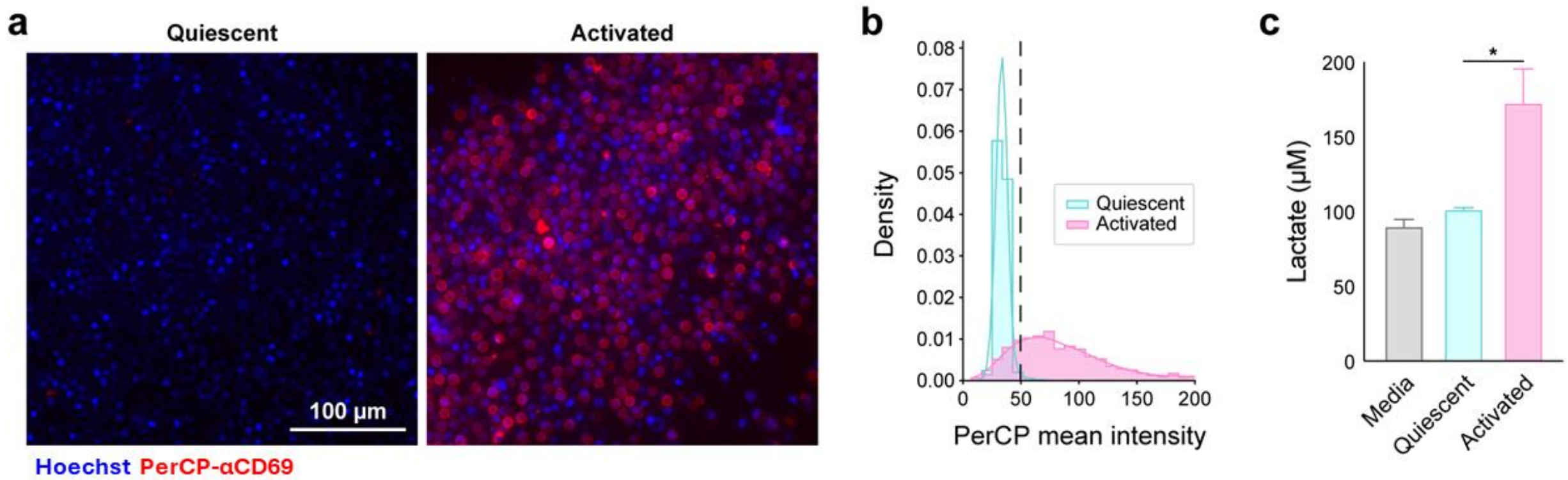

Supplemental Figure 5. 24-hour quiescent and PMA/Ionomycin-activated PBMCs. (a) Representative Hoechst (blue) and αCD69 (red) staining. (b) Quantification of single-cell staining reveals 79.6% PerCP-αCD69 expression after 24 hours in activating conditions. Threshold defined at 1.5 standard deviations over the mean PerCP-αCD69 intensity of the quiescent group. (c) Lactate production after 24 hours was increased by 70% with activation compared to quiescent. n= 3153 activated and 1839 quiescent PBMCs. *P<0.05, two-tailed unpaired T-test.

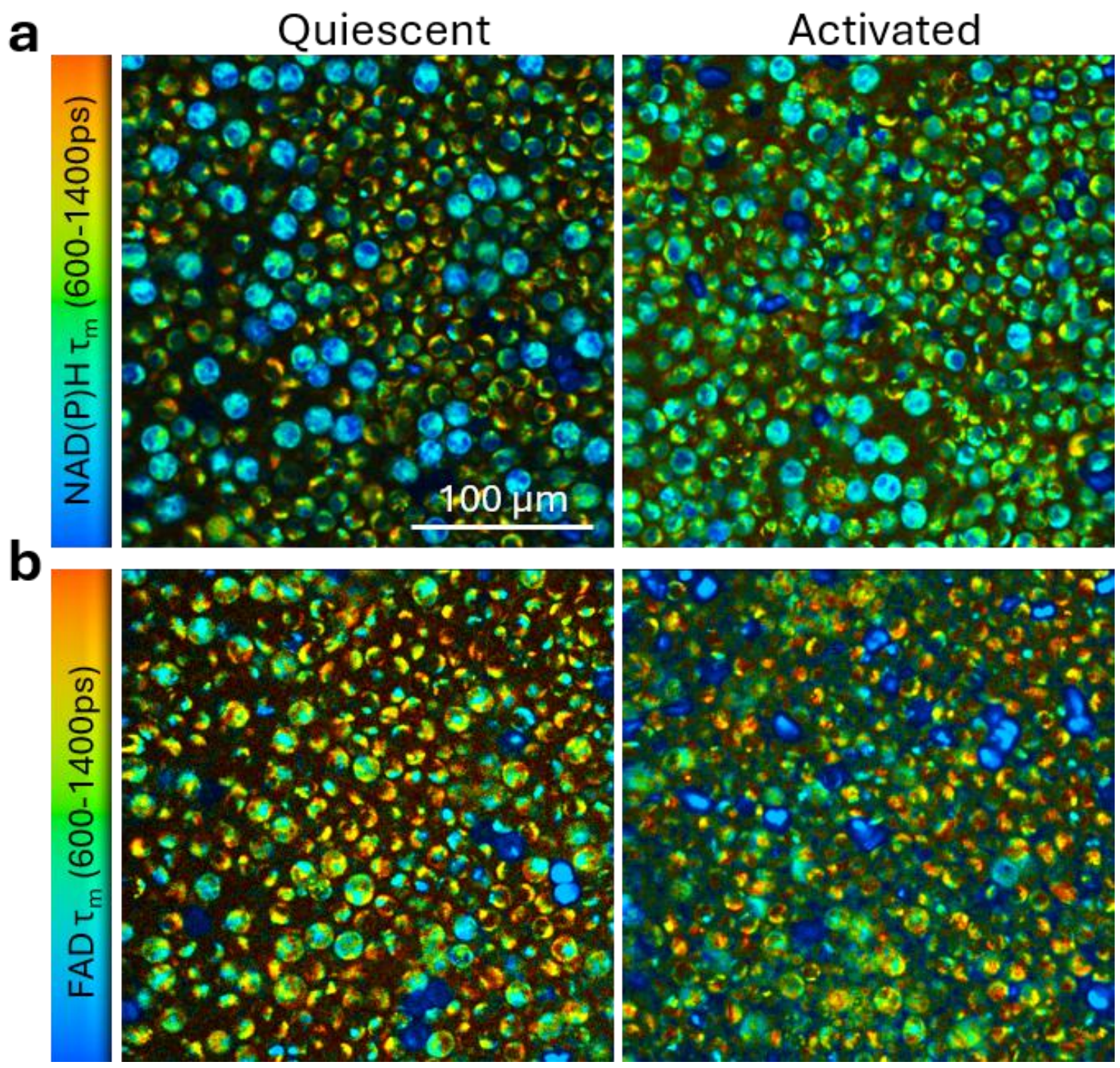

Supplemental Figure 6. Two-hour quiescent and PMA/Ionomycin-activated PBMCs. Representative images of unmasked quiescent and activated PBMC cell populations for NAD(P)H τ_m (a) and FAD τ_m (b). The bright short lifetime red blood cells and debris were removed from analysis using an NAD(P)H τ_1 filter as described in methods.

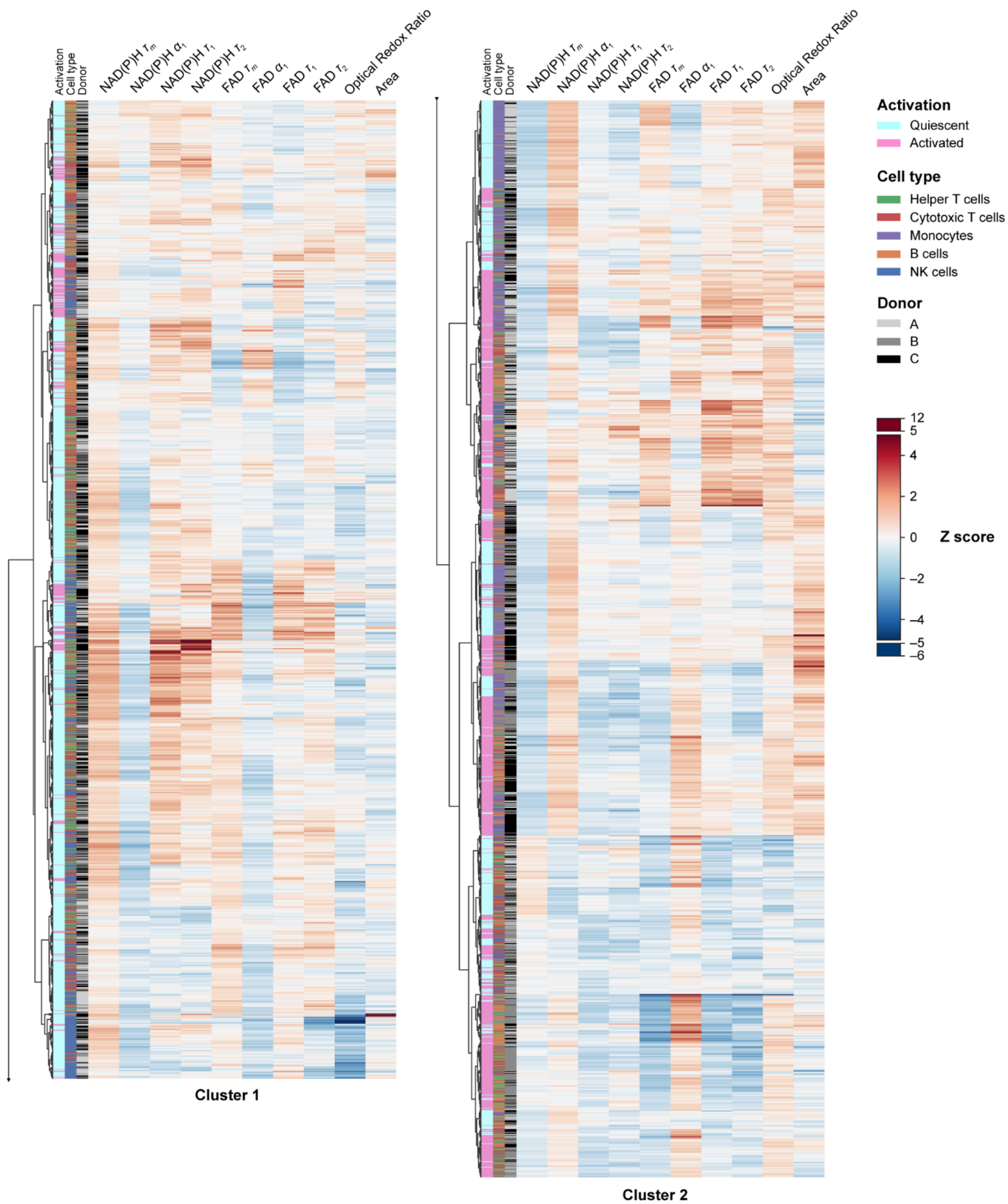

Supplemental Figure 7. Hierarchical clustering of all PBMCs. Heatmap and hierarchical clustering of single-cells was calculated based on the z-scores (the difference between cell mean and population mean divided by the population standard deviation) of 10 OMI variables (NAD(P)H and FAD τ_m, τ_1, τ_2, α_1, the ORR, and cell area). Cell counts by donor, cell type, and activation can be found in supplemental table 2.

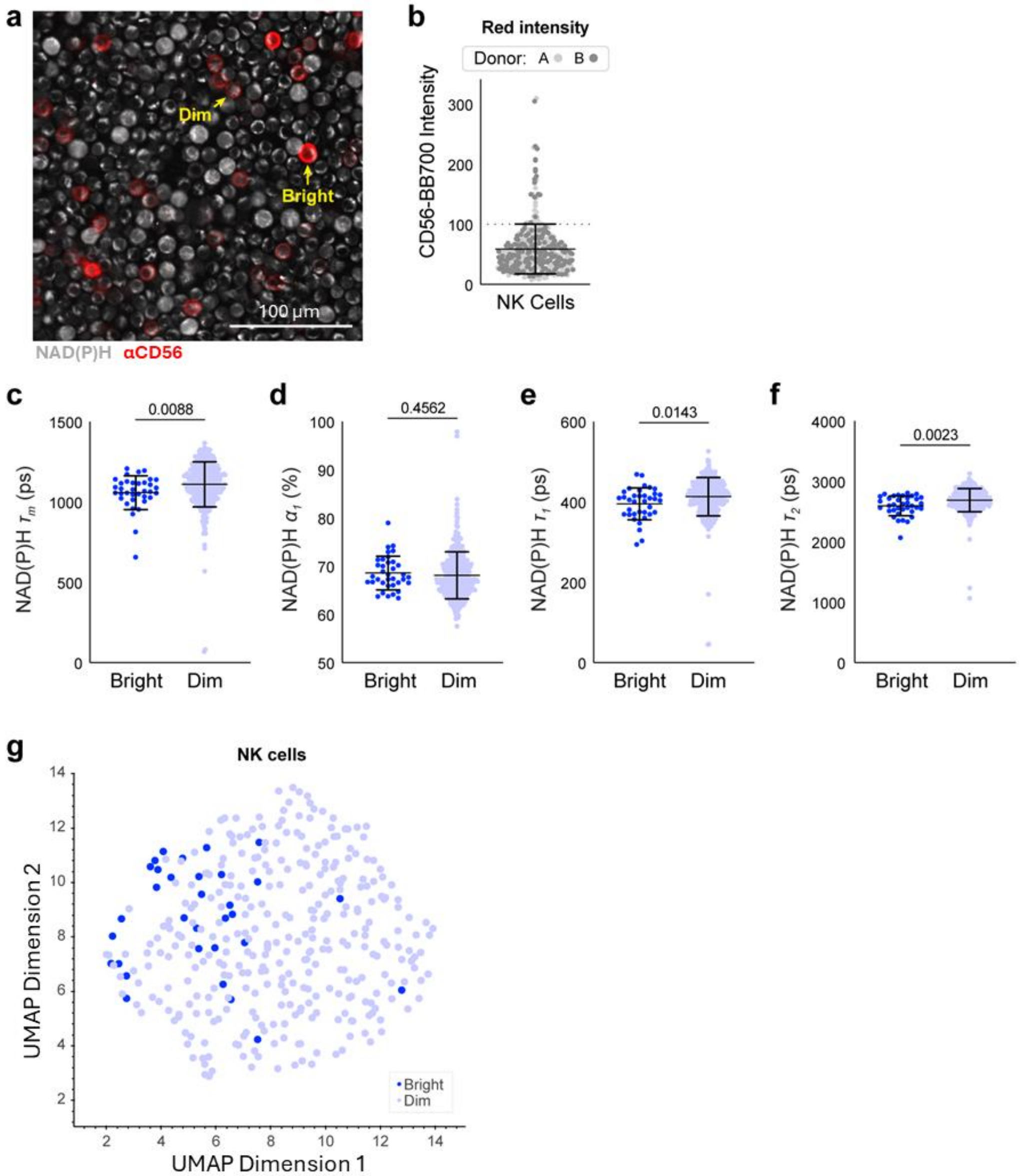

Supplemental Figure 8. Metabolic subtypes within the quiescent NK cell population. (a) Representative αCD56 staining (red) overlaid on NAD(P)H intensity (grayscale). (b) An αCD56 intensity value at 100 was selected such that approximately the top 10% brightest CD56-NK cells fell above the threshold. (c-f) NAD(P)H lifetime values showed some significant differences using a two-tailed unpaired t-test. Data given as mean and standard deviation overlaid on single-cell plots. (g) UMAP shows clustering of CD56 bright and dim NK cells using all OMI variables (NAD(P)H and FAD τ_m, τ_1, τ_2, and α_1, the ORR, and cell area). n = 327 quiescent NK cells.

Target	*Isotype*	*Clone*	*Reactivity*	*Fluorophore*	*Catalog #*	*Brand*	*Lot*	*Concentration*
CD4	Mouse IgG1, κ	SK3	Human	PerCP-Cyanine5.5	344607	Biolegend	B380254	100 µg/mL
CD8	Mouse IgG1, κ	SK1	Human	PerCP-Cyanine5.5	344709	Biolegend	B356155	50 µg/mL
CD14	Mouse IgG2a, κ	M5E2	Human	PerCP	301847	Biolegend	B415977	400 µg/mL
CD19	Mouse IgG1, κ	LT19	Human	Star Bright Blue 700	MCA1940SBB700	Bio-Rad	100004436	1.0 mg/ml
CD56	Mouse IgG2b, κ	NCAM 16	Human	Brilliant Blue 700	566574	BD Horizon	3226480	125µg/mL
CD69	Mouse IgG1, κ	FN50	Human	PerCP	310927	Biolegend	B290414	200 µg/mL

Supplemental Table 1. Details of primary antibodies including the host species, clone, manufacturer, and catalog numbers.

Cell type	*Donor*	*Quiescent*	*Activated*
Helper T cells	A	188	78
Helper T cells	B	161	153
Helper T cells	C	204	132
Cytotoxic T cells	A	160	46
Cytotoxic T cells	B	257	135
Cytotoxic T cells	C	137	172
Monocytes	A	159	95
Monocytes	B	150	82
Monocytes	C	116	128
B cells	A	115	110
B cells	B	116	66
B cells	C	137	164
NK cells	A	170	129
NK cells	B	157	18
NK cells	C	183	120

Supplemental Table 2. Cell counts by cell type, activation, and donor across all experiments.
